# Slow march to surgery: using the COM-B model to explore the lived experience of individuals with knee osteoarthritis, awaiting joint replacement surgery

**DOI:** 10.1093/rap/rkag100

**Published:** 2026-08-08

**Authors:** Nathan Brookes, Yeliz Prior, Stephen Preece, Jennifer Parker, Simone Battista

**Affiliations:** Centre for Human Movement and Rehabilitation, School of Health and Society, The University of Salford, Manchester, UK; Physiotherapy Department, Northern Care Alliance NHS Foundation Trust, Salford, UK; Centre for Human Movement and Rehabilitation, School of Health and Society, The University of Salford, Manchester, UK; Centre for Human Movement and Rehabilitation, School of Health and Society, The University of Salford, Manchester, UK; Centre for Human Movement and Rehabilitation, School of Health and Society, The University of Salford, Manchester, UK; Centre for Human Movement and Rehabilitation, School of Health and Society, The University of Salford, Manchester, UK

**Keywords:** knee osteoarthritis, total knee replacement, qualitative research, lived experience, non-surgical management, health beliefs, coping strategies, healthcare interactions, COM-B model, behaviour change

## Abstract

**Objectives:**

This study aimed to explore the lived experiences of people with knee osteoarthritis (KOA) awaiting Total Knee Replacement (TKR).

**Methods:**

Fifteen semi-structured interviews were conducted with participants with a diagnosis of KOA. Transcripts were first analysed inductively with reflexive thematic analysis. Inductive themes were deductively aligned to the COM-B model (‘Capability’, ‘Opportunity’, ‘Motivation’ and ‘Behaviour’) to provide additional understanding of how people navigate the management of KOA and their experiences while awaiting surgery.

**Results:**

Four inductive themes were generated and mapped into the COM-B component. (1) ‘Altered health beliefs drive a biomedical focus on KOA management’ (Capability): participants largely understood KOA as an inevitably degenerative condition culminating in surgery. (2) ‘Interactions with healthcare professionals reinforce surgical inevitability’ (Opportunity): participants described clinical encounters that strengthened their biomedical interpretations and shaped expectations of surgery as the primary solution. (3) ‘The emotional burden of KOA’ (Motivation): emotional strain contributed to decisions to pursue surgery. (4) ‘Coping strategies for managing KOA pain’ (Behaviour): participants employed a range of self-directed strategies to manage symptoms while awaiting surgery, often with limited guidance.

**Conclusion:**

This study provides a novel, theory-informed understanding of how people awaiting TKR make sense of, engage with, and manage KOA. Using the COM-B model to map these experiences illustrates how current pathways can unintentionally weaken motivation and opportunity to engage with evidence-based first-line interventions like exercise. These findings indicate the value of supporting people with KOA to better understand and access first-line options before considering surgery.

Key messages

- Biomedical beliefs influence the conceptualisation of knee osteoarthritis and may reduce perceived capability for self-management.
- Inconsistent interactions with the healthcare system undermine engagement with first-line, guideline-recommended interventions.
- Reduced quality of life, affected by declining wellbeing and function, influences individuals’ motivation for knee replacement surgery.

## Introduction

Knee osteoarthritis (KOA) is common and affects around 16% of the global population [1]. Symptoms such as pain, swelling and stiffness often lead to loss of function and reduced quality of life [2]. Although non-surgical interventions, such as education, exercise and weight management, are recommended as first-line management strategies [3, 4], many people with KOA ultimately consider total knee replacement (TKR), when symptoms persist [3, 5]. TKR has a high overall satisfaction rate [6, 7], but outcomes vary. Up to 30% of people report no clinically significant improvements in their symptoms after 1 year [6], and the procedure carries risks [8] that make it unsuitable for up to 25% of people with KOA [9, 10]. Moreover, following the COVID-19 pandemic, waiting times for TKR surgery remain high [11], and the rate of TKR surgery continues to increase [12].

Despite these considerations, many people do not engage with, or are not supported in engaging with, first-line interventions before deciding to proceed to surgical consultations [3, 4]. Understanding why this occurs requires insight not only into clinical outcomes but also into how people make sense of their condition, access care and navigate treatment decisions in everyday life. Previous systematic reviews have synthesised studies related to the lived experience of people with KOA. However, no reviews have been published in the post-COVID era, a period during which multiple KOA guidelines have been updated [3, 4].

A 2013 review by Smith *et al*. [13] included qualitative studies which focused on living with hip and knee OA. More recently, Wride and Bannigan [14] reviewed nine articles related to knee pain, although three of the included studies were not specific to KOA. Both reviews [13, 14] identified themes related to functional decline, challenges with understanding the condition and negative experiences of managing the condition. In contrast, a review by Wallis *et al*. [15] exclusively focused on KOA, analysing 21 qualitative studies, involving 665 participants. They identified seven major themes and concluded that patient-centred approaches that address psychosocial factors may support self-management. Further research is required to understand the lived experience of people with KOA within a post-COVID healthcare system. Specifically, how increased reliance on telehealth [16] and long waiting times for surgery may have impacted the management of KOA.

The aim of our qualitative study was to explore the experiences of living with KOA in individuals who felt they had exhausted non-surgical management and were waiting for a TKR. Specifically, we wanted to understand how life had changed since diagnosis and the effect of KOA on function, work life and social life. Moreover, we aimed to understand individual beliefs about what was causing their pain, what interventions they had previously received and what brought them to opt for surgery.

## Methods

### Study design

We conducted a qualitative interview study and analysed the transcripts using reflexive thematic analysis (RTA) [17]. Themes were developed inductively and subsequently clustered within the COM-B model [18] to facilitate interpretation of challenges in self-managing KOA and the factors that influenced a desire for surgery. The COM-B model was chosen since it is a widely validated behaviour change framework that accounts for the multifactorial and varied experiences of people with KOA [18].

Ethical approval was received from the University of Salford Research, Enterprise, and Engagement Ethical Approval Panel (ID: 7000) and Health Research Authority and Health and Care Research Wales Approval Committee (22/YH/023). The study followed the ‘Declaration of Helsinki’ and is reported in line with the ‘Consolidated Criteria for Reporting Research for reporting qualitative studies’ (COREQ) [19].

### Participants

Participants with relevant experience, specifically those on a waiting list for TKR, were purposefully recruited. This population was chosen because they were at the end of their care, and they were supposed to try all non-surgical interventions before the option for surgery. Eligible participants were identified via mailouts coordinated by clinical research nurses at local hospital trusts in the North West of England. In parallel, a targeted Facebook social media campaign was launched. The research team was contacted by interested individuals via telephone or email. In total, 114 individuals were screened for eligibility, of whom 10 were identified through the Facebook campaign. Research staff provided detailed information about the study, screened participants for eligibility, and obtained written informed consent. Eligibility criteria included: age over 45, a clinical diagnosis of KOA, persistent knee pain for at least 6 months, currently on an orthopaedic waiting list for TKR, and sufficient English proficiency to understand the participant information sheet and consent process. Of the 15 participants consented, eight were recruited from the NHS and seven via the Facebook campaign. Following consent, demographic information was collected and the interview was scheduled.

The sample size was determined using the ‘information power’ model [20]. Given our focused aim, the specific sample, a purposive sampling approach, and our expertise in qualitative research, we estimated an appropriate sample size of 13 to 16 participants.

### Data collection

The semi-structured interview topic guide was designed by the research team and the project’s Patient and Public Involvement and Engagement (PPIE) group. The topic guide aimed to support participants in reflecting on their experiences of KOA, including diagnosis, interventions, well-being and waiting for surgery. The interview topic guide is presented in Table 1.

**Table 1:** Semi-structured interview topic guide.

Interview questions for patient users
**Section 1: General experiences of living with knee osteoarthritis**
Q1. Can you tell me about your experience of living with knee osteoarthritis? *(prompts: what has changed? what is the effect on function, work life, social life?)*
**Section 2: Beliefs about knee osteoarthritis**
I’m keen to hear about your beliefs surrounding knee osteoarthritis. Q1. What do you understand is causing your knee osteoarthritis pain? Q2. What treatment have you had for your knee osteoarthritis pain? Q3. What treatment do you believe will resolve your knee osteoarthritis pain? What have you been recommended?
**Section 3: Finish**
Q1. Do you have anything else you would like to tell me about your experiences of living with knee osteoarthritis?

The following topics/questions may be discussed. The qualitative work will remain flexible with respect to participants’ agendas but we will cover the broad topics/questions noted. It is common in qualitative work to iteratively develop topics and questions as new ideas emerge from early data collection. Therefore, we may add new topics as the interviews progress and data collection continues.

All interviews were conducted over the telephone or via Microsoft Teams between December 2022 and October 2023 individually by two interviewers (Y.P. and N.B.), who created field notes to aid in memory and recall of the interview. All interviews were conducted 1:1, and the interviewers did not know the participant prior to the interview. No repeat interviews were completed. All interviews were audio-recorded by Teams and transcribed verbatim by an independent transcription company using manual transcription methods. The transcripts were not returned to the participants, but they were checked for accuracy by the study team. Manual revision of these transcripts was undertaken by N.B., with missing or unclear sentences completed where possible based on contextual cues and field notes. These transcripts were included in the analysis with appropriate caution, and their limitations were considered during theme development. No identifiable information was included in the transcriptions, and all participants were assigned pseudonyms.

### Data analysis

We conducted a two-step approach to analyse the qualitative dataset. Themes were first analysed inductively using Reflexive Thematic Analysis (RTA) by Braun and Clarke [21]. RTA is a flexible qualitative method that allows for the identification of patterns within data, while acknowledging the active role of the researcher in theme development. The analysis was conducted following the six steps (Table 2) of RTA within a constructionist paradigm, adopting an experience-driven perspective, and employing semantic-level coding to ensure themes were grounded in participants’ lived experiences [21, 22]. Two researchers (Y.P. and N.B.) independently read the transcripts in detail, and N.B. coded the text (i.e. open coding). An initial set of themes was developed through the interpretation of the data by the study team. A reflexive coding framework was developed to support theme refinement, with codes and illustrative quotes iteratively reviewed and adjusted. The process is reported in Table 2.

**Table 2:** Six steps of thematic analysis.

Phases	Process	Authors’ involvement	Authors’ actions
1. Data familiarisation	N.B. and Y.P. read and re-read the transcripts for familiarity. N.B. noted down keywords to aid in the development of codes	N.B. and Y.P. took part in this stage	Kept record of all transcripts and field notes. Spent time engaging with the transcripts. Keywords noted.
2. Coding	N.B. developed initial codes from included quotes and discussed the codes with Y.P.	N.B. and Y.P. took part in this stage	A record of the codes and the generation of coding was kept. Audit trail of code generation. Meeting notes kept to understand authors’ thought process related to coding.
3. Generating initial themes	Y.P., N.B., S.P., J.P. and S.B. generated initial themes from the codes	Y.P., N.B., S.P., J.P. and S.B. took part in theme development	Initial themes noted with process of combining themes recorded to ensure a transparent process
4. Reviewing and refining themes	N.B. reviewed the themes, discarding some until a consistent set of final themes were presented	Y.P. oversaw the process. N.B. discussed the final themes with Y.P., S.P., J.P. and S.B.	Y.P., N.B., S.P., J.P. and S.B. decided on the final themes and ensured conciseness, including themes that reflected the data
5. Defining and naming themes	N.B. reviewed each theme, defined its context and finalized the theme names	S.B. reviewed the process and met with N.B. to discuss the theme context and theme names	Y.P., N.B., S.P., J.P. and S.B. agreed on the final theme names
6. Producing the report	N.B. drafted the report following a meeting with all authors	Y.P. and S.P. reviewed the first draft. All authors reviewed subsequent drafts	N.B. completed the final draft of the report

N.B. is a physiotherapist and honorary research fellow in physiotherapy and identifies as male. Y.P. is an occupational therapist, PhD and professor of clinical rehabilitation and identified herself as female. S.P. is a Biomechanist, PhD and professor of biomechanics and identified himself as male. J.P. is a clinical trial manager, PhD and identified herself as female. S.B. is a physiotherapist, PhD and research fellow and identified himself as male. All researchers are interested in osteoarthritis and have experience in RMD and conducting qualitative research.

Following inductive theme development, we applied the COM-B model as an interpretive deductive framework to map each theme to its dominant behavioural component (Capability, Opportunity, Motivation, Behaviour). COM-B offers a coherent behavioural framework that integrates complex influences and highlights modifiable factors that could inform future interventions. This strengthens the explanatory power of the analysis and supports the translation of qualitative findings into practical, behaviour-change-focused recommendations. Deductive mapping was undertaken by N.B. and reviewed by the study team. Inductive data (participant quotes) related to each theme were subsequently deductively mapped to the relevant COM-B model component. Where a quote could plausibly align with more than one COM-B component, all components were listed. Finally, each inductive theme was considered in context and grouped under the COM-B component that provided the most conceptually relevant explanatory fit (see Supplementary Table S1, available at *Rheumatology Advances in Practice* Online).

The research team included clinicians and qualitative researchers with experience in musculoskeletal health. The interviewers were trained in qualitative interviewing and were familiar with the clinical context of knee osteoarthritis but did not have a therapeutic relationship with the participants. To address the potential of professional background shaping data interpretation, emerging interpretations were discussed collaboratively, reflected on assumptions throughout the analytic process, and grounded theme development in the data.

### Patient and public involvement

Two PPIE contributors with lived experience of knee osteoarthritis reviewed the interview topic guide and provided feedback on the content, wording and accessibility of questions. Their input helped ensure that the interviews used patient-centred language and addressed issues relevant to people living with KOA. PPIE input was summarised using the GRIPP 2 Reporting Checklist [23] (See Supplementary Table S1, available at *Rheumatology Advances in Practice* Online).

## Results

The final sample consisted of 15 participants (8 men, 7 women) aged 64–81 years, who had been on an orthopaedic waiting list for a TKR for an average of 8 months. The interview length ranged from 12 to 43 min.

Four primary themes and seven subthemes were identified from the inductive thematic analysis and are presented in Table 3, alongside illustrative quotes.

**Table 3:** Primary themes and sub-themes with illustrative quotes.

Theme/sub-theme	Illustrative Quotes
**Theme 1: Altered Health Beliefs Drive a Biomedical Focus on KOA Management** **Sub-theme 1: Surgery as the Only Long-Term Option**	“I was just walking along, well, walking into the house and my knee went. Er, that’s five months ago, and I’ve had it’s just like bone-on-bone pain and it doesn’t matter what, erm, you know, er, painkillers I have, it doesn’t relieve it at all.” (‘ZAHIDA’ TKRB03) “I have the bone on me bone…with no cartilage in between at all. That’s completely wore out. And that’s on the right knee and on the left knee, I have a little bit of cartilage left but that’s wearing out. So I, I kind of understand what’s going on and why it’s got to this point.” (‘MARIE’ TKRB12) “I’ve seen the x-ray myself over, over the year, and you can see that, and it’s been pointed out to me, and it is deteriorating.” (‘MICHAEL’ TKR08) “Well, um, all I can think is that, that what’s happening as a result of the arthritis is that the, the kind of, um, lubrication in the knee joint, er, is drying up and my bones, er, sort of grind together, and, that, that, that comes up through the nervous system.” (‘DAVID’ TKRB11) “It will for, well, I’ve, I assume it will and I mean I’ve, I’ve spoken to lots and lots of people that have had the operation, and you know, they, they, they say it makes a big difference.” (‘ROBERT’ TKRB04) “Well, I can’t think of anything else. I mean, I, I, I sort of say, look, I really want the operation.” (‘DAVID’ TKRB11)
**Theme 2: Interactions with Healthcare Professionals: Focus on a Surgical Solution** **Sub-theme 1:** **Biomedical Framing by Healthcare Professionals** **Sub-theme 2: Physiotherapy: Inconsistent and Often Ineffective**	“I saw the, erm, the surgeon a couple of weeks ago, he said keyhole surgery’s not an option with mine because it’s, it’s arthritic bone and it’s bone on bone, you know, so it wouldn’t do any good. He said all, all that would do is disturb all the debris that’s behind my knee…I just, I just got the feeling off him—he didn’t say it, but I just got the feeling, well, you know, what do you expect at your age? It’s wear and tear, you know…” (‘ZAHIDA’ TKRB03) “So they put, they put me on his list. He said it needed a complete replacement due to the fact that it was down to the bone on the left hand knee and also that the right knee was in need of being replaced.” (‘SHONA’ TKR513) “Er, it was, erm, the physiotherapist gave me some, I think it was about four exercises to do, er, which he said that would strengthen the knee, erm, ready, ha, ready for the operation sort of! And er, he said, when you, you know, if you have the operation or when you have the operation, you’ll probably come back and see me again.” (‘BRIAN’ TKRB01’) “In my left knee. It was my GP who said around the January time, just before we had lockdowns, we need to get you on a waiting list now because the gap between injections with pain relief was getting less and less.” (‘MAUREEN’ TKRB07) “Alright, and getting difficult to walk. So they put, they put me on his list. He said it needed a complete replacement due to the fact that it was down to the bone on the left hand knee and also that the right knee was in need of being replaced aswell.” (‘SHONA’ TKR513) “I’ve not, I’ve not managed to get to physiotherapy, but I really would—you know, I was hoping that this would have started a few weeks ago, so, um—but if I can get some kind of physiotherapy to help me, you know, keep going.” (‘SARA’ TKRB06) “I’ve just done a bit of physio last week in Stepping Hill, which seems to have made it more worse, and the reason being, she asked me to do certain physio things, and since doing them, my actual knee itself has gone worse than what it was, so whether the muscles are not compensating enough for the bone, or the bone is not accepting the muscle thingy, I’m not quite sure.” (‘MALCOLM’ TKR417)
**Theme 3: The Emotional Burden of Living with KOA** **Sub-theme 1: Valued Social Engagement Amid Physical Constraints** **Sub-theme 2: Pain-Related Sleep Disruption and Its Ripple Effects**	“I jokingly said to the doctor that I saw, the first doctor, er, first consultant that, ‘I’m gonna end up in a nursing home at this rate, because it’s taking so long. And he just said not to be so silly. Er, it’s been a year, and I’m still messing about, and I said the only other alternative is to throw myself down stairs.” (‘BRENDA’ TKRB02) “It has a massive impact, really, and it has a massive impact on your mental health. I mean, I’m only 65, 64. So really, I shouldn’t be like this. I should be…another 20 years, 15 years, I should be like this. And it’s awful.” (‘MARIE’ TKRB12) “And, and I’m, I’m not fit now. And, um, I, I didn’t expect that I would be here because of this…osteoarthritis and, and whatever else is going on in my knee. Oh, it is incredibly frustrating. And, and there are times when it, it does get to you and you feel I wouldn’t say to-, depression, but you feel down. You feel down and then you have a better day. Um, but my, my activities, my ability to do things has been, it’s been—it’s, they’ve stopped. They’ve stopped."(‘LUCY’ TKRB05) “And I said, and I just said to him, Well, I’m sorry, but it’s taken my whole quality of life. I can’t do anything. I’m a keen outdoor person. I, I like gardening and everything, so it’s stopped me doing any of that.” (‘FRANCES’ TKRB03) “Erm, so, er, my world closed down rather quite a lot. Me and my partner are—were big walkers, always have been. Erm, so from doing ten miles on a walk throughout a day, you know, with breaks, erm, that got worse and worse to where I’m, I was down to two miles during all this period.” (‘MAUREEN’ TKRB07) “So it, it’s always there. So, so what I find is at night, the pain in both of my knees, it feels as though my knees are on fire and somebody is playing a steel drum inside them.” (‘LUCY’ TKRB05) “And the biggest issue for me as well. Well, one of the biggest is lack of sleep. Because it goes stiff in bed, and then, obviously, we move about a lot in the night, don’t we? And th-, then I wake up, every time I turn and move, I wake up because it hurts.” (‘MAUREEN’ TKRB07) “It’s more when you’re lying down. I’m all right when I first go to bed, and I have a cushion which I put between my legs now to separate my knees, which helps, but then again, around about 3:30, 4:00 o‘clock, the pain kicks in. I put a bit of stuff on, a bit of cream on it, and then hope that goes away, but sometimes it just carries on, and then you’re tossing and turning.” (‘MALCOLM’ TKRB17)
**Theme 4: Coping Strategies for managing KOA pain** **Sub-theme 1: Adapt or Avoid** **Sub-theme 2: Pain Relief Strategies**	“I’m still active, and I’ve always been active. Er, what the, the knee has done, erm, it’s obviously curtailed to a large extent walking. Erm, I do a lot of outdoor activities and, and of course, it’s, it’s impinged on that.” (‘RON’ TKRB10) “If I want to turn around, I’ll walk around on my, on my foot in effect, as opposed to just twisting my body. So I’m very careful of that. Erm, and also, I’m careful when, er, sort of, er, stepping down a bit of a distance ’cause I always go down with the other leg.” (‘BRIAN’ TKRB01') “I’m really struggling. Not so much just sitting around but if, if I’m doing any walking, especially slow walking around the shops or just wondering about, then that tends to make it worse, as does just standing. So, you know, I try to, to sit.” (‘ROBERT’ TKRB04) “I used to be very active, erm, I used to do jogging and various things along those lines and try and keep fit and, and, and various sports, but unfortunately, over the last ten years, erm, gradually I’m, I’m not—I mean, even with the grandkids now, I find it difficult, they want me to play football with them and run with them, er, and, and, and, again, it’s, it’s very painful to actually do that.” ('JAMIE’ TKRB08) “And in the last week or so I’ve noticed that the, um, the side of my, the inside of my right knee is, is starting to bother me driving. I mean I’ve never had that yet, so, but I don’t go very far because I can’t walk around the shops. You know, I can’t walk around the Trafford Centre, because that would be too much for me. So I don’t drive far.” (‘LUCY’ TKRB05) “Yeah, steroid, er, cortisone. Yeah, yeah. I’ve had, er, two injections in my knee, which, erm, haven’t, basically haven’t made any difference.” (‘BRIAN’ TKRB01) “I’ve been given a leg brace, which, what happens with the leg brace, it digs into the side of your leg. So then I stopped using it all together now, because it’s not doing much good.” (‘MALCOLM’ TKRB17) “My doctor put me on co-dydramol, er, and I was in so much discomfort that I started taking ibuprofen, aswell, but my stomach’s not in good condition, and, er, I really shouldn’t have been taking them, but I’m managing, at the moment. I’m taking two co-dydramol in the morning, and two, um, ibuprofen, and at, sort of, four/five o‘clock, round the early teatime.” ('BRENDA’ TKRB02)

The themes and sub-themes were grouped under a relevant COM-B component (see Supplementary Table S2, available at *Rheumatology Advances in Practice* Online).

### Capability

Capability refers to an individual’s psychological and physical capacity to engage in a behaviour. Altered health beliefs about KOA reinforced a biomedical solution for KOA and a focus on surgery as the primary option of choice. These beliefs appeared to shape how participants viewed their capacity to self-manage. Therefore, under this COM-B component, we clustered one primary theme and its related sub-theme, which explained how participants made sense of KOA and the role of surgery in their care.

#### Theme 1: Altered health beliefs drive a biomedical focus on KOA management

Participants commonly interpreted that the cause of their pain was due to ongoing degeneration, often described as ‘wear and tear’ linked to the ageing process. This belief provided a framework for understanding their symptoms, with many expressing that the loss of cartilage and the wearing out of the joint made sense as explanations for their pain. The concept of ‘bone-on-bone’ contact was particularly significant because it was considered a clear indicator of severity and progression. The loss of joint space was interpreted as evidence of worsening disease. This was often represented and confirmed through radiographic imaging and clinical interpretation.

##### Subtheme 1: Surgery as the only long-term option

In this context, surgery emerged as a central and often inevitable solution. Many participants viewed surgery as the only viable option to improve pain and restore quality of life. There was a strong positive expectation surrounding surgical outcomes, often influenced by communication with others who had undergone the procedure or prior experiences of surgery on the other limb. Some participants felt that their outcome depended on the surgeon, highlighting a passive role in their care. However, participants also acknowledged that not everyone manages well post-operatively and that challenges may persist. Additionally, some anticipated the belief of future surgical needs, such as a TKR on the other knee because of disease progression. Overall, participants’ beliefs about degeneration and imaging findings shaped their understanding of OA and framed surgery as a necessary and logical next step, often overshadowing consideration of non-surgical management strategies. This reduced their perceived capability to self-manage the condition.

### Opportunity

Opportunity refers to external factors that make a behaviour possible or prompt it. This included social opportunities such as advice or support from others and physical opportunities such as access to services. Therefore, under this COM-B component, we clustered one primary theme and two related subthemes, which explained the external factors that affected their opportunity to self-manage KOA.

#### Theme 2: Interactions with healthcare professionals: focus on a surgical solution

Participants described varied and often contrasting experiences with the main healthcare professionals involved in managing knee OA, namely General Practitioners (GPs), orthopaedic surgeons and physiotherapists. Participants reported a consistent message from healthcare professionals that KOA was progressive and would, at some point, require surgical intervention. However, this often led to a sense of frustration when participants experienced delays to surgery or poor communication from healthcare services about waiting lists and surgical dates. Overall, the role of non-surgical management was interpreted as a stop-gap, while waiting for surgery.

##### Subtheme 1: Biomedical framing by healthcare professionals

Interactions with medical professionals were frequently framed around the inevitability of surgery, with many participants reporting that a TKR was presented as the only viable option. This definitive framing often followed imaging or clinical assessments, contributing to a sense of irreversible damage. The language used by clinicians and their role as ‘experts’ reinforced beliefs about degeneration and validated concerns about worsening symptoms.

##### Subtheme 2: Physiotherapy: inconsistent and often ineffective

While physiotherapy was generally viewed as acceptable, participants interpreted it as a short-term intervention to prepare for surgery and to maintain mobility, rather than a tool for long-term management or prevention. This framing may have limited opportunities for behaviour change and reduced the perceived value of physiotherapy as a standalone intervention.

Several participants described physiotherapy input as inconsistent and disjointed, with exercises that were either too basic or too challenging. A lack of personalisation was a common concern, with some feeling that the exercises did not reflect their prior experience or knowledge of physical activity. Moreover, pain during physiotherapy was a recurring theme.

A few participants reported benefit from specific modalities such as hydrotherapy, though these were seen as less effective as the condition progressed. Finally, in response to dissatisfaction with physiotherapy, some participants turned to online resources to guide their exercise routines. However, this self-directed approach was accompanied by uncertainty about the appropriateness and safety of the exercises.

Overall, interactions with surgeons, GPs and physiotherapists shaped participants’ beliefs about their condition and influenced their treatment expectations. The dominant narrative of surgery as inevitable often overshadowed the potential of non-surgical management, leaving participants feeling passive in their care and uncertain about alternative options.

### Motivation

Motivation refers to the internal processes that influence decision-making and behaviour. This includes reflective processes such as planning or evaluation and automatic processes such as emotional responses or habits. Therefore, under this COM-B component, we clustered one primary theme and two related subthemes. The themes explain the factors which demotivate participants to self-manage their condition namely their experience of disruption of valued activities and the effect on their emotional well-being.

#### Theme 3: The emotional burden of living with KOA

The emotional toll of KOA was expressed by many participants, who reported feelings of frustration, sadness and anxiety. This was particularly evident in relation to a reduction in their capability to engage in valued activities. Participants described a decline in mood and mental health, noting changes in personality and emotional resilience due to persistent pain.

##### Subtheme 1: Valued social engagement amid physical constraints

Despite ongoing pain, many participants expressed a strong desire to remain active and socially engaged, though this often required significant adjustments. For example, participants often modified how they walked (e.g. distance, location, support) or how they engaged in daily routines. These adaptations were framed as necessary responses to the complexity of their condition. Activities such as going on holiday or watching grandchildren play sport were still pursued, albeit with reduced intensity and increased planning around rest breaks. Overall, there was a sense of trial and error as participants navigated these limitations, striving to preserve valued aspects of life, despite physical constraints. This was often framed as putting on a brave face amid significant challenges and ongoing frustration.

##### Subtheme 2: Pain-related sleep disruption and its ripple effects

Sleep disturbances were commonly reported, often linked to night pain. While some participants found relief through medication or positional adjustments (e.g. using a pillow between their legs), others described poor sleep as a significant contributor to emotional distress and reduced quality of life/daily functioning. Despite challenges, some participants maintained a comparative perspective, noting that their condition was not as severe as others. This framing often coexisted with a health-conscious mindset and a reluctance to rely on medication, though many acknowledged its role in maintaining function and sleep quality.

Overall, participants’ well-being and social engagement were reduced as the condition progressed. This was related to ongoing pain and a loss of function, which affected many participants’ ability to interact with valued activities. Poor sleep quality affected well-being, and, overall, a vicious cycle emerged in which well-being, pain and social engagement were negatively affected.

### Behaviour

Behaviour refers to the observable actions or decisions an individual takes in response to their condition or situation. Therefore, under this COM-B component, we clustered one primary theme and two subthemes. The themes explain the observable actions taken by individuals to self-manage and how this changed over time.

#### Theme 4: Coping strategies for managing KOA pain

Participants reported a range of non-surgical strategies to manage their pain and maintain daily functioning, with varying degrees of success. All participants described an initial active coping style, often seeking out support for their condition when newly diagnosed. However, as pain progressed, non-surgical care was unavailable, not effective or perceived as not useful, and perceived controllability diminished; motivation to actively cope reduced. As such, many participants perceived surgery as their remaining option and consequently reported greater reliance on passive coping strategies.

##### Subtheme 1: Adapt or avoid

Participants changed their approach to their condition over time. They commonly adapted or avoided physical activities. Walking for pleasure, navigating stairs, shopping and driving were all activities that participants reduced or ceased, often citing pain, stiffness or the effort required. Exercise was important for some, with those attending the gym modifying their programmes to reduce knee pain. However, others reported stopping the gym altogether due to their symptoms.

##### Subtheme 2: Pain relief strategies

Pain relief strategies varied widely. Common medications included paracetamol, ibuprofen, co-codamol, co-dydramol, naproxen and tramadol. While some participants found these helpful, others reported limited effectiveness or adverse side effects such as grogginess and constipation. A few participants expressed reluctance to rely on medication but acknowledged its necessity.

Non-pharmacological aids such as elasticated and rigid braces were trialled, with mixed results. Some participants experienced swelling or discomfort, while others used braces selectively (e.g. for longer walks) with modest relief. Crutches were adopted by some to reduce walking-related discomfort, though they introduced new challenges in daily functioning.

Experiences with steroid injections were similarly mixed. While some participants initially benefitted, others reported diminishing returns or no improvement over time. Overall, participants used a range of active and passive coping strategies to manage their pain. Although some strategies were helpful, many participants recognised a decline in their function, while their pain continued to increase.

## Discussion

This study provides novel insights into the lived experience of individuals with KOA in the post-COVID landscape. Applying the COM-B model provided explanatory depth, situating our results. COM-B helped illustrate how Capability, Opportunity, and Motivation interact to shape Behaviour. For example, altered health beliefs (Capability) were reinforced by healthcare interactions (Opportunity), which amplified emotional burden (Motivation) and drove a shift from active coping strategies to passive behaviours, such as reliance on medication and prioritising surgery (Behaviour). This interplay highlights that decisions regarding KOA management are not linear but emerge from interconnected biopsychosocial influences. Using COM-B, therefore, moved the analysis from ‘what people experience’ to ‘why these behaviours occur’, offering a framework for identifying modifiable targets for intervention.

A key finding in our study was the dominance of biomedical beliefs about pain, which affected the participants’ ability to self-manage and to rationalise non-surgical care. Despite advances in understanding the multifactorial nature of OA [24] and the poor correlation between radiographic findings and pain [25], many participants in our study viewed KOA as a degenerative process with limited non-surgical management options. Although OA is no longer considered a wear-and-tear condition [24, 26], this outdated view was often reinforced by imaging findings and clinician language, particularly phrases such as ‘bone-on-bone’ or ‘wear and tear’. These results are consistent with international studies that have revealed comparable narratives about how the use of inappropriate or negative language to describe OA can shape patient perceptions and decisions [15, 27, 28].

In the UK context, these beliefs are also reinforced by system-level factors that create a disconnect between guidelines and patient experiences. Participants described inconsistent access to physiotherapy and limited engagement with other non-surgical management options, which are considered first-line interventions (e.g. exercise). In fact, non-surgical care was often viewed as preparatory rather than a standalone option for KOA management. This aligns with previous international research that found that patients rarely view non-surgical care as sufficient, and access to allied health professionals is inadequate [29]. Although influenced by the reductions in healthcare access due to COVID-19, findings underscore the need for system-level changes. Specifically, improved access to non-surgical care, which enables repeated engagement in a broad range of evidence-based options throughout their healthcare journey.

Previous research has described the emotional challenges of living with KOA [30]. In our study, many participants described frustration, anxiety and low mood, which affected their motivation to self-manage. Both NICE [3] and EULAR [4] guidelines endorse integrated care packages that combine exercise with psychological strategies. In addition, EULAR recommends individualized psychological input such as Cognitive Behavioural Therapy (CBT) [4]. However, unlike non-surgical chronic low back pain pathways, which stratify care [31], KOA pathways rarely feature systematic stratification. The absence of such stratification may contribute to the underutilisation of tailored interventions. This is despite evidence that integrating pain-coping skills and behaviour change into standard care yields small-to-medium improvements in pain and function at 3–6 months [32].

Previous research has explored the interrelationships between coping behaviours and OA severity [33], with a shift from active to passive coping, over time. Many participants reported a transition from topical pain relief or paracetamol to opioids such as tramadol. This is concerning as opioids worsen baseline KOA pain over time [34], lead to faster progression of KOA [34] and are not recommended by some OA guidelines [5]. While opioid prescription is lower in the UK than in the USA [35], concerns about overuse remain [36]. In addition, many participants in our study reported attempts to mitigate their pain experience by reducing physical/social activity. This is problematic as the importance of pre-operative health and well-being on post-operative recovery [37] is well known. Although prehabilitation and ‘waiting well’ initiatives are recommended, there is considerable variance in what is offered [38]. This is of concern as returning to activity and social interactions are key post-surgery recovery goals for people undergoing TKR [39].

Overall, the participants lived experience of KOA was influenced by internal and external factors which affected their capability, opportunity and motivation to self-manage. These findings underscore the importance of ongoing, accessible interventions that promote active coping, including education, peer support and exercise.

### Study limitations

There are limitations to the study. Firstly, our findings were specific to the NHS England healthcare system and, as such, may not be generalisable to other regions. However, other work within international healthcare systems reports similar themes [40, 41]. Secondly, we did not formally investigate the influence of other informal sources of information, such as social media, on the participants’ lived experiences. As such, we are unable to make recommendations beyond the healthcare discourse. Thirdly, we did not collect detailed demographic information such as ethnicity, which limits the ability to assess diversity within the sample. This is particularly important for understanding the experiences of underserved groups. Finally, the sample represents a specific subgroup of people with KOA who had progressed to consideration of surgery. Their experiences, beliefs and management strategies may differ from those of individuals who are earlier in their care pathway or who are successfully managing their condition, without surgery.

## Conclusion

This study illustrates how key themes related to the lived experience of KOA are interconnected and influence how individuals navigate their condition. By applying the COM-B model, we offer insight into how peoples’ beliefs, healthcare interactions and emotional well-being may collectively shape (non)engagement with first-line non-surgical management and contribute to perceptions of surgery as a necessary step. These findings suggest that supporting people with KOA through consistent, equitable evidence-based interventions may help strengthen self-management and broaden treatment expectations. Such approaches may, in turn, help ensure that decisions about surgery are more informed and preference-based, an important consideration given rising rates of TKR surgery and increasing pressure on healthcare resources.

## Supplementary Material

rkag100_Supplementary_Data

## Data Availability

Anonymised individual participant data will be made available following any reasonable request made to the corresponding author, subject to a data sharing agreement and UK research governance regulations.
