# Supplementary material for: Slow march to surgery: using the COM-B model to explore the lived experience of individuals with knee osteoarthritis, awaiting joint replacement surgery
Source: Rheumatol Adv Pract. 2026 Aug 8;10(3):rkag100. doi: 10.1093/rap/rkag100 (PMC13509947; doi:10.1093/rap/rkag100)
Supplement: Supplementary file 1 [file rkag100_supplementary_data.zip › Supplementary table S1- Gripp PPIE.docx]

| **GRIPP2 reporting item** | **Description** |
| --- | --- |
| 1. Aim | As part of this study, the group advised on the interview topic guide which was used to interview participants reported in this study. |
| 2. Methods | Two PPIE members with KOA contributed to the interview topic guide review. JC worked with the research team as a lead PPIE member for a range of studies over the past 8 years. WM also worked with the research team on a range of KOA projects since 2018. They are both retired professionals, who identify as White British, living in Greater Manchester. We conducted a pretrial PPIE meeting whereby they read the interview topic guide and provided feedback related to the wording of questions. |
| 3. Study results (outcomes) | The PPIE group successfully contributed to the trial methodology. Positive outcomes included: (1) the review of a patient-centred interview topic guide. |
| 4. Discussion and conclusions (outcomes) | PPIE contributed to this aspect of the study by providing the experience of perspectives of living with KOA and guidance on the use of language for ‘lay’ people. This allowed us to review the interview topic guide, simplifying the terms and language used as required. |
| 5. Reflections/critical perspective | We consider the input from the PPIE team as positive. Challenges included the PPIE members working on a similar trial and as such, having less time to focus on this study. This meant they were unable to contribute to other aspects such as thematic analysis. Nevertheless, where they did provide expertise, provided a richness of lived experience to our methodology. |
