# Supplementary material for: Slow march to surgery: using the COM-B model to explore the lived experience of individuals with knee osteoarthritis, awaiting joint replacement surgery
Source: Rheumatol Adv Pract. 2026 Aug 8;10(3):rkag100. doi: 10.1093/rap/rkag100 (PMC13509947; doi:10.1093/rap/rkag100)
Supplement: Supplementary file 1 [file rkag100_supplementary_data.zip › Supplementary table S2- Primary themes, sub-themes and included quotes.docx]

| Capability | The individual’s psychological and physical capacity to engage in the behaviour. This includes having the necessary knowledge, skills, and physical ability. | References to understanding (or misunderstanding) of the condition, treatment, or symptoms; physical limitations (e.g., pain, mobility issues); or cognitive challenges. |
| --- | --- | --- |
| Opportunity | External factors that make the behaviour possible or prompt it. This includes physical opportunities (e.g., access to services) and social opportunities (e.g., support from others). | Mentions of healthcare access, waiting times, referrals, interactions with professionals, or environmental/social support or barriers. |
| Motivation | The internal processes that influence decision-making and behaviour. This includes reflective processes (e.g., planning, evaluating) and automatic processes (e.g., emotional responses, habits). | Expressions of desire, intention, hope, fear, frustration, or reasoning behind decisions (e.g., choosing surgery for better quality of life). |
| Behaviour | The observable actions or decisions taken by the individual in response to their condition or situation. | Descriptions of what the participant actually did (e.g., stopped taking medication, sought private care, waited for results, accepted surgery). |

Definition of COM-B categories

| **Code name/s** | **Code**  **description** | **Coded data** | **Key experiences and behaviours in participants’ accounts** | **Mapping to COM-B model** |
| --- | --- | --- | --- | --- |
| **Theme:** **Altered Health Beliefs Drive a Biomedical Focus on KOA Management** | | | | |
| **Codes** | | | | |
| **Investigations**  **Cause- wear and tear of joint and loss of cartilage** | Participants interpretations of investigations and how they influenced health beliefs  Participants interpretation of the cause of their pain | I've had, I've had another x-ray, and I was told then that it would be up to three or four weeks before it came, and I thought probably the best thing for me to do is to wait until I get the report. I guess it will be copied to me, or, or I can get it from my GP anyway, um, and, um, when I've got all that information, I can sort of ring up and say, 'Look, er, in the light of this, can you give me a date for the, for the operation?' TKRB11:  Erm, I've had, erm, several MRI scans and X-rays over a period of time. Erm, I mean, I, I looked up yesterday that, erm, I had some tablets for my knee in 1995 and later on in 2010. But I didn't finish taking all the tablets, so I presume it was only, erm, sort of a temporary occurrence of a joint ache. Er, I may have banged it or something, so, er, I was, I was prescribed those tablets in those years. And then, erm, since then, I've had an MRI scan, erm, and an X-ray, and the latest X-ray or scan, I can't remember, erm, the surgeon was recommending I needed a replacement knee, er, so I'm on the waiting list, but that might be about six months away anyway TKRB01 ( also coded in healthcare interactions- communication with GP/surgeon)  The knee is so bad I, that it's bone on bone. That was, er, confirmed by an x-ray last December (TKRB02) (Also coded in cause 2- bone on bone)  'Well, we'll wait for the, erm, MRI scan results to come back,' he said, 'and then I'll refer you to a different hospital.' He said, 'You know, obviously it's not the right…place for you,' you know. So we got the results back and he's referred me to, erm, Sheffield Claremont…and the man, you know, the consultant there was totally different. Very, very helpful, explained everything, and he just said there is no other treatment at all that they can offer except a full knee replacement or leave it as it is’ TKRB03 (also coded in healthcare interactions- communication with GP/surgeon)  We used, we used to live up in Aberdeenshire and, and the process of sort of establishing what it actually was started up there with the physiotherapy do-, department and I had some x-rays done, both knees and hips, which indicated that I had osteoarthritis in, in my knees and hips to varying degrees. Since then., which will be about, oh, eight years ago, nine years ago, it's gradually got worse to the point where, you know, I'm really struggling. TKRB04  ..because I went through the GP and I was hoping to, to have an MRI scan on it, er, if I, if I, if it hadn't happened by now, I was then going to go and get the private one to find out what on earth is going on…TKRB05  or even before that. I went to the GP about the pain in it, in the knee, who said, 'We'll get it scanned,' and one thing and another. So we had all that done and they decided that it's osteoarthritis so it needs replacing TKRB12 (also coded in healthcare interactions- communication with surgeon)  Well, not really. Just when they looked at pictures said it was bone on bone, they did actually. TKR513  -the, the first, first operation on the knee that they've offered me is a replacement. The surgeon looked at the x-rays and said, 'There's nothing I can do apart from replace it.' So...I was a bit shocked myself because I thought they were just going to, you know - there was a bit of floating bit and then they do this, and he just went, 'There's nothing I can do with that apart from replace it.' TKRB08: (also coded in healthcare interactions- communication with surgeon)  Yeah, definitely, because the osteoarthritis has progressed. So on the x-ray you can see that the two bones that meet through the top and bottom, that they, they have lost the gap between the first x-ray and the second x-ray TKRB05 (also coded in cause- bone on bone)  No, that was, that's it, that was the only option. She said there was no oth- when the x-ray showed up, she said there was no other alternative, but surgery. TKRBO2 (also coded in communication with GP/surgeon)  Yeah, I think - to be honest with you, I've - because of the, the - I've, I've seen the x-ray myself over, over the year, and you can see that, and it's been pointed out to me, and it is deteriorating. So, it's just a question of, well, do I have it now, erm, while, while they operate, or do, do I want to carry on a stage, and I just thought, well, erm, to be honest with you, I, I had serious health issues about three years ago and I thought to myself, well, the quality of life, I might as well take a better quality of life now and have surgery.. TKRB08 (Also coded in TKR expectations)  I've not been told, really. I'm presuming it's old age and wear and tear was what my GP said, but the consultants didn't really say TKRB07  gradually over the years, with wear and tear, I've got the osteoarthritis in the knee because of it TKRB08 | Participant expecting the results of the scan will suggest an operation is required  Participants Interpretation was that from the results of the scan the surgeon has made their recommendations. Knee OA pain/ severity can be determined by scan. Lack of control to self-manage.  Participant interprets that a scan confirms the presence of bone on bone and the severity of symptoms.  Participant interprets that based on the MRI results, there isn’t anything that can be done other than surgery  Linking investigation which demonstrates osteoarthritis with worsening function/pain  Belief that a scan will explain what is going on in the body  Osteoarthritis is diagnosed from a scan and pain is linked to scan findings, hence needing a knee replacement  Explanations from healthcare professionals reinforced view that pain was related to bone on bone changes  In contrast, surgeons interpretation of scan results were unsettling as believed keyhole surgery would be offered  The xray results prove to the participant that the osteoarthritis has progressed  The participant perceived that the xray informed the surgeons decision regarding OA management  Xray informing participants decision about OA management  Interpreted wear and tear was the cause but remained uncertain. Relating this to age  Linking together wear and tear with osteoarthritis and relating this to age | Capability  Capability  opportunity  Capability  Capability  opportunity  Capability  Capability  Capability  opportunity  Capability  Capability  opportunity  Capability  Capability  opportunity  Capability  Capability  Capability |

| **Cause- bone on bone and loss of cartilage**  **Expectations of surgery**  **Lack of control** | Participants interpretation or understanding of the cause of their pain  Sub-theme 1- Surgery as the Only Long-Term Option | it was probably just, er, gen-, degeneration. It was to be expected but, erm, after one or two visits to the, to the doctor, erm, when they mentioned osteoarthritis, erm, that combined with the symptoms changing and getting worse, erm, that convinced me then that I was in the throes of knee problems. TKRB10 (Also coded to communication with surgeon)  with no cartilage in between at all. That's completely wore out. And that's on the right knee and on the left knee, I have a little bit of cartilage left but that's wearing out. So I, I kind of understand what's going on and why it's got to this point TKRB12  Now, what I can gather is this stuff you have in between your knee, like a cushion-y thing, it's wear and tear, so what happens is, that wears off, and then your bones start to… which is maybe what's happened, and this is causing the pain. TKR417  It is. I mean, I spoke to the consultant before and she was saying that it is the mechanical side to the whole thing because now I have the bone on me bone…with no cartilage in between at all. That's completely wore out. And that's on the right knee and on the left knee, I have a little bit of cartilage left but that's wearing out. So I, I kind of understand what's going on and why it's got to this point TKRB12 (Also coded in cause 2- bone on bone)  Er, it's where the joint is and the supporting cartilage between the two surfaces, surfaces. So more in the way of generally causing a bone-on-bone, erm, problem. TKRB01  The knee is so bad I, that it's bone on bone. TKRB02  Yeah, definitely, because the osteoarthritis has progressed. So on the x-ray you can see that the two bones that meet through the top and bottom, that they, they have lost the gap between the first x-ray and the second x-ray. TKRB05 (also coded in investigations)  Erm, as far as I know it's deterioration of the bone on bone and, and perhaps the cartilage. TKRB09  Well, um, all I can think is that, that what's happening as a result of the arthritis is that the, the kind of, um, lubrication in the knee joint, er, is drying up and my bones, er, sort of grind together. TKRB11  It is. I mean, I spoke to the consultant before and she was saying that it is the mechanical side to the whole thing because now I have the bone on me bone…with no cartilage in between at all. That's completely wore out. And that's on the right knee and on the left knee, I have a little bit of cartilage left but that's wearing out. So I, I kind of understand what's going on and why it's got to this point TKRB12 (Also coded in wear and tear)  Erm, well I was offered, erm, well, I'm saying offered - I was told that it could probably be flushed out but, er, when I saw the, erm, the surgeon a couple of weeks ago, he said it's not an option with mine because it's, it's arthritic bone and it's bone on bone, you know, so it wouldn't do any good. He said all, all that would do is disturb all the debris that's behind my knee and cause more problems. TKRB03 (Also coded in healthcare interactions- nocebo)  Alright, and getting difficult to walk. So they put, they put me on his list. He said it needed a complete replacement due to the fact that it was down to the bone on the left hand knee and also that the right knee was in need of being replaced aswell TKR513 (Also coded in healthcare interactions- communication with GP/surgeon)  Er, well, my, erm, my wife had one seven years ago, erm, and she's been fine with hers. Erm, I mean, you hear of people who - you hear of people who have had the replacement knee and they're, they're in a lot of pain, er, and other people have had no problems at all. The wife had hers seven years ago, erm, and now she's got a problem with the other knee. But the replacement knee has been brilliant really. TKRB01  It will for, well, I've, I assume it will and I mean I've, I've spoken to lots and lots of people that have had the operation, and you know, they, they, they say it makes a big difference TKRB04  Well, I'm hoping that a knee replacement will help the situation and the pain that I'm currently having. If it doesn't, because with osteoarthritis you never get rid of that, do you? So it, it's always there.  TKRB05  Um, so I don't know. I have looked at having an operation. If I could get it to sort of - when I say that, I'm reluctant, because I think sometimes you're, you're messing up something that you could perhaps improve yourself, which is why, when they suggested physiotherapy and, err, you know, this course, I thought, ooh, that could be, that could be really good for me. Yes, that, that's the way I think. It should be the last resort TKRB06  Some have been very pleased, the majority. Some haven't been overly pleased, erm, not because it hurts them so much. I think it's about movement. A knee op hurts more than a hip op, when you're doing your recovery period, erm, the pain is worse. Well, I'm not mad about the pain, erm, and I understand that I have to do it to, erm, get my leg to a 90-degree angle and maybe even back further than that. TKRB07  Yeah, I think - to be honest with you, I've - because of the, the - I've, I've seen the x-ray myself over, over the year, and you can see that, and it's been pointed out to me, and it is deteriorating. So, it's just a question of, well, do I have it now, erm, while, while they operate, or do, do I want to carry on a stage, and I just thought, well, erm, to be honest with you, I, I had serious health issues about three years ago and I thought to myself, well, the quality of life, I might as well take a better quality of life now and have surgery.. TKRB08 (Also coded in investigations)  I'm going next week for a replacement…Erm, yeah, I mean, er, I'm, I'm looking forward to it in the respect that it, it's hopefully, erm, the only way that I can rid myself of the pain and, erm, maybe get back to something like what I would consider my normal way of life. I've, I've looked at various things, I've spoken to people who have had knee replacements and, erm, I, I would say, like a lot of people, erm, my confidence in what's going on in the NHS, er, isn't as high as it might be, so I, I, just feel that if the, if the surgeon in charge can do a decent job, then, er, it's as much as I can expect really TKRB10  Well, I can't think of anything else. I mean, I, I, I sort of say, look, I really want the operation TKRB11  I think after they do the first knee, I'm gonna have to wait now and get the other knee done. And if it - the time span is the same as this one, it's gonna be forever. So I, I try not to think…too far ahead because I just get disappointed…I spoke to quite a few people who's had the knee operation and they said, 'You're - it's great. You know, after a couple of weeks, months, you're fine.' TKRB12  I’ve travelled. Around the world. And I’m wanting. To go away again. But I’m, I’m waiting for a time when I’m pain free. Well not. Pain free but less so. I’m looking forward to it TKR416  Well, I mean, not really. I'd just like to repeat the point that I really would like to get the operation done. I mean…I, I am going to, um, MRI, the, er, medical clinic, quite soon. I mean, I, I, I could look up the date, but I think it's in, in early November. Um, and, er, I, I'm going to ask them, you know, okay, um, can you sort of make the point to your colleagues in Salford, Salford Royal that I really am, you know, very keen to go and have the operation, because I just want, you know, to have a fuller life. TKRB11  Um, no, I've not. I've, I've seen a couple - well where I walk, because everybody sees me walking the dog, there was a chap, he had two new, two new knees done at the same time and because he couldn't walk at all, and then when I saw him, he said, 'Oh.' He feels so much better because he's able to walk. But I, I don't really know anybody who's had knee replacements. And you always get the, um, 'Oh, it didn't work for me.' 'Yes, it worked for me.' 'No, it didn't.' And, and I sorta thought, well, it is gonna work.  Because I know that, that, that my head, in my head it's going to work because I want my life back. TKRB05  It's so painful. It interferes with everything, just about everything, and it just gets progressively worse and more painful. TKRB12  Well, I mean, yeah, there, there's, there's nothing, erm, that will, will rid me of the inconvenience and the pain, erm, other, I feel, than having the mechanics of it sorted because I, I feel it's - it can only possibly It, it can only possibly get worse as, as more and more of it wears away, erm, and because I can't do, erm, significant exercises to strengthen the muscles around it, erm, er, I think it's just a downward path. TKRB10  I was diagnosed with me left knee. That's got arthritis as well. So that's gonna need another knee replacement. TKRB12    Erm, that, that the pain - I mean, sometimes, I'll go for a week and it's, it's relatively brilliant. I can, I can, I can walk almost normally, and then other times, like the past two or three weeks, it's been absolutely awful and, and just simple things, getting up off the chair, er, sitting down, going to the toilet even, it, it, it's, it's an effort. It's a real effort… it's my first experience of any kind of, erm, er, something being beyond my control. Er, I've never been ill at all for anything, so this is completely a, a new experience for me, and I've always - I wouldn't say I've taken life for granted, but I've always, erm… I just sort of face what's in front of me for the day and do it, you know.It's, er… And it's when you can't do it that I suppose it str-, it strikes home then.TKRB10  Well, actually more or less I've sort of, um, been rather passive and have sort of, um, taken what I've been given. TKRB11 | Belief that pain is related to degeneration  Belief that symptoms are linked to the wearing out of the knee and a lack of cartilage and that this makes sense.  Belief that pain is related to degeneration  Belief that symptoms are linked to the wearing out of the knee and a lack of cartilage and that this makes sense.  Belief that pain is related to bone on bone  Bone on bone seen as an indicator of the severity of condition  The loss of space between the bones is an indicator of progression  Belief that pain is related to bone on bone. Less certain about involvement of cartilage  Belief that pain is related to changes in the joint with bone contact  Belief that symptoms are linked to the wearing out of the knee and a lack of cartilage and that this makes sense.  Bone on bone seen as an indicator of the severity of condition  Surgery required due to lack of cartilage and joint has degenerated to bone  Overall positive expectation of surgery. Recognises that not everyone manages well.  Expecting surgery to improve pain. Reinforced from communication with others  Hopeful that surgery will improve symptoms and unsure of how to change pain due to nature of osteoarthritis.  Recognising that surgery should be the last resort and their may be capacity to improve pain.  Overall positive expectation of surgery. Recognises that not everyone manages well. Understands that post op there will be challenges  Viewing surgery as the option which will improve quality of life  Viewing surgery as the only option to improve pain and quality of life. Focus on surgeon being responsible for the outcome.  Viewing surgery as the only option and has a strong desire to go ahead  Influenced by others, surgery is viewed as helpful. Predicting the need for a TKR on other knee.  Viewing surgery as the option which will improve quality of life. Recognises that pain may continue after surgery.  Viewing surgery as the option which will improve quality of life  Recognising there may be challenges but viewing surgery as the option which will improve quality of life    The participant described frustration in relation to the the condition progressing and becoming more painful.  Participant had lost hope in improving the condition and felt that it would progress over time  Participant linking a new diagnosis with progression to surgery  Participant struggling with th up and down nature of the condition and felt that it was beyond their control to manage which was a new experience  Participant honestly describing approach to condition as passive | Capability  opportunity  Capability  Capability  Capability  Capability  Capability  Capability  Capability  Capability  Capability  Capability  opportunity  Capability  Capability  Capability  Motivation  Capability  Motivation  Capability  Motivation  Capability  Capability  Motivation  Capability  Capability  Motivation  Capability  Capability  Motivation  Capability  Capability  Capability  Capability  Motivation  Capability  Motivation  Capability  Capability  Behaviour |
| --- | --- | --- | --- | --- |
| Theme 1- Altered Health Beliefs summary narrative  Participants commonly interpreted their knee osteoarthritis as a result of degeneration, often described as "wear and tear" and linked to aging. This belief provided a framework for understanding their symptoms, with many expressing that the loss of cartilage and the wearing out of the joint made sense as explanations for their pain. The concept of bone-on-bone contact was particularly salient, viewed as a clear indicator of severity and progression, often confirmed through imaging such as X-rays or MRIs. The loss of joint space was also interpreted as evidence of worsening disease.  Pain was consistently linked to these structural changes, especially bone-on-bone contact, though some participants were less certain about the specific role of cartilage.  In this context, surgery emerged as a central and often inevitable solution. Many participants viewed surgery—particularly total knee replacement (TKR)—as the only viable option to improve pain and restore quality of life. There was a strong positive expectation surrounding surgical outcomes, often influenced by communication with others who had undergone the procedure. However, participants also acknowledged that not everyone manages well post-operatively and that challenges may persist.  Sub-theme 1- Surgery as the Only Long-Term Option  Some expressed hopefulness that surgery would alleviate symptoms, while others saw it as a last resort, recognising that there might be capacity to improve pain through other means. A few participants placed significant responsibility on the surgeon for the outcome, highlighting a passive role in their own recovery. Additionally, some anticipated future surgical needs, such as a TKR on the other knee, based on their current experience and beliefs about disease progression.  Overall, participants’ beliefs about degeneration and imaging findings shaped their understanding of OA and framed surgery as a necessary and logical next step, often overshadowing consideration of non-surgical or conservative management strategies. | | | | |
|  | | | | |
| **Theme 2: Interactions with Healthcare Professionals: Focus on a Surgical Solution** | | | | |
| **Codes**  **Physiotherapy** | Sub-theme 1: Biomedical Framing by Healthcare Professionals  Sub-theme 2: Physiotherapy: Inconsistent and Often Ineffective | Er, it was, erm, the physiotherapist gave me some, I think it was about four exercises to do, er, which he said that would strengthen the knee, erm, ready, ha, ready for the operation sort of! And er, he said, when you, you know, if you have the operation or when you have the operation, you'll probably come back and see me again. TKRB01  I've had physiotherapy, um, which she, at that point, the young lady that I saw said my left knee was worse than the right, but, um, the exercises they wanted me to do, I was fine with, but then they tried to put me on a, a more severe exercise regime, and I couldn't cope with it. Because, er, I, I was carrying too much weight, and I said if I could come off the list for physio for a while, while I got some weight down. It didn't, didn't work out quite the way I expected it to. They just took me off and left me without anything. TKRB02 | Communication with the physiotherapist reinforced the view that the role of exercise was focused on preparation for surgery. This focus reduced the opportunity for behaviour change.  Physiotherapy viewed as acceptable however once the exercises were progressed, they were deemed too challenging. As such, the participant was discharged and no longer supported. | **Opportunity**  **Opportunity** |

|  |  | I, I had a year of physio. Well, them giving me exercises, er, it, it wasn't, um, month-by-month. It was a coupla mon-, and then would be a coupla months after that. But it was giving me exercises and showing me how to move my leg and what I should be doing. You know, how to step, put your heel down, put your foot, your toe down then.. it was just, it was simple things, but because I have been fitter than I am now, um, I, I'm very muscular, my, my body is able to do, to do more, but I wasn't given anything advanced. So what they were giving to me was, was put your leg straight and move your toes towards the body.I, I couldn't of - yeah. I, and I can do that, and I have looked on the internet with lots of different things. Done, I'd seen there's, er, things that people will recommend, I've seen things that don't recommend. You, you know, so it's a bit of a minefield. So you have to go with what you are capable of doing. So it, and it, again, I, I, I think physio, nor-, normal physio like that, was a waste of time on me. TKRB05  Um, do you know, because I, I was actually diagnosed or, or referred during lockdown, I had a telephone conversation with somebody who, basically, gave me some exercises over the phone, which really wasn't very good, was it? Not even - nothing. Not even on an email or anything. Because I was trying - I was literally trying to, err, work out what he was telling me to do, which was, basically, um, almost like my knee bent stood up at a 45 degree angle, and I had to sort of say, well, how do I..So I've never ever been given a piece of paper with a picture of somebody doing an exercise, or seen anybody. TKRB06:  I've not, I've not managed to get to physiotherapy, but I really would - you know, I was hoping that this would have started a few weeks ago, so, um - but if I can get some kind of physiotherapy to help me, you know, keep going, um, until such time TKRB06  I went to a physio talk at Stepping Hill, er, last week, erm, which scared me a bit. I've been told to do these exercises, I - when I went to this talk, which I'm doing pre-op, even though they're for post-op because it'll strengthen your muscles. But, if it come - say to myself, but being a - having been a walker for a, a long time, I've probably got fairly strong leg muscles anyway. And… But, I-, I've been doing them a bit since I've c-, came back last week, erm, because the lass who did that talk, that was physio lady. She, erm, recommended you to start doing it be-… You know, before you're op. So I'm doing as I'm told, erm, but it hurts me to do it, I'll be honest. To try and lie me leg out flat on the bed with a little towel under me foot hurts.Er, but I'm doing it, so - because they say, h-, h-, he told me it hurts more. A knee op hurts more than a hip op, when you're doing your recovery period, erm, the pain is worse. Well, I'm not mad about the pain, erm, and I understand that I have to do it to, erm, get my leg to a 90-degree angle and maybe even back further than that. TKRB07  Over the, over the, yeah, over the, over the years, I've, I've, I've received, er, physiotherapy, erm, er, and I also did one of the early studies that Nathan, er, was involved in about three, four years ago where they gave us exercises to do and, and, and then various things like that to get the muscles and, and stuff. So I’ve carried on, carried on trying, trying to do that, and I think that, that helped out substantially to, er, limit the, er, the discomfort. Yeah, to be honest with you, the, the - they used to - to be honest with you, after it, a day, a day or two after it, it was, it was like unbearable... the pain, because you'd, you'd done the exercise. But then, erm, it - what was daft was once that initial, initial shock kind of wore off, you had got that bit more mobility and that, and, and it wasn't as painful. So, there is an initial, it'd be like shock treatment thing on it, but then I think the more you do it, the more it kind of wears off. I think it, yeah, it was that - it was kind of to help, erm, help, help reduce, erm, the pain and also increase mobility within the knee because... the other thing I've got is, is, is restriction in, in the, erm, flex, flexibility of the knee, to be honest with you. TKRB08  Er, yes, I've had, er, the various, erm, events of physiotherapy, erm, and quite honestly, it's made absolutely no difference whatsoever.Yeah, some exercises and, erm, you know, the, the, the rubber bands and that kind of thing. I mean, I've always been - er, because I've always been interested in sport, I, I, I've a, I've a fair idea of what the muscles do…And which muscles I'm using, so, erm, I, I knew straightaway that it, it wasn't actually doing anything for me. TKRB10  I have seen them all. I have been to the - at Hope, they have, like, a warm pool where you can do physio in the warm pool. I have, I've done that. They've give me, erm, a gym, er, membership that - I did that for quite some time. I've been to the physios, endless physio appointments and exercises, so I've done that. I think the warm pool - that helped for quite some time. But then that stopped helping. Everything seems to have a time limit attached to it. Now, maybe this is because your knee advances further and it's just not enough. TKR417  From it I did do some exercises but they weren’t helping, they hurt most of the time. The whole 6 week course was well ran and meant to help me. The fact was that no, I didn't like doing it. TKR513  Subtheme 1: Biomedical Framing by Healthcare Professionals  **Communication with GP/Surgeon**  Well, we'll wait for the, erm, MRI scan results to come back,' he said, 'and then I'll refer you to a different hospital.' He said, 'You know, obviously it's not the right…place for you,' you know. So we got the results back and he's referred me to, erm, Sheffield Claremont…and the man, you know, the consultant there was totally different. Very, very helpful, explained everything, and he just said there is no other treatment at all that they can offer except a full knee replacement or leave it as it is’ TKRB03 (also coded in altered health beliefs- investigations)  I went to the GP about the pain in it, in the knee, who said, 'We'll get it scanned,' and one thing and another. So we had all that done and they decided that it's osteoarthritis so it needs replacing TKRB12 (also coded in altered health beliefs- investigations)  Alright, and getting difficult to walk. So they put, they put me on his list. He said it needed a complete replacement due to the fact that it was down to the bone on the left hand knee and also that the right knee was in need of being replaced aswell TKR513 (Also coded in altered health- beliefs cause 2- bone on bone)  Erm, I've had, erm, several MRI scans and X-rays over a period of time. Erm, I mean, I, I looked up yesterday that, erm, I had some tablets for my knee in 1995 and later on in 2010. But I didn't finish taking all the tablets, so I presume it was only, erm, sort of a temporary occurrence of a joint ache. Er, I may have banged it or something, so, er, I was, I was prescribed those tablets in those years. And then, erm, since then, I've had an MRI scan, erm, and an X-ray, and the latest X-ray or scan, I can't remember, erm, the surgeon was recommending I needed a replacement knee, er, so I'm on the waiting list, but that might be about six months away anyway TKRB01 ( also coded in healthcare interactions- communication with GP/surgeon)  No, that was, that's it, that was the only option. She said there was no oth- when the x-ray showed up, she said there was no other alternative, but surgery. TKRBO2 (also coded in communication with GP/surgeon)  or even before that. I went to the GP about the pain in it, in the knee, who said, 'We'll get it scanned,' and one thing and another. So we had all that done and they decided that it's osteoarthritis so it needs replacing TKRB12 (also coded in altered health beliefs- investigations)  And they thought it was a, a meniscus tear, and they did operate on that initially, but said that it was too, um, it was frayed. They couldn't put it back together and there was a ramp lesion that they didn't see the point of fixing. So. But in this process, they did notice that there was osteoarthritis. So, so the osteoarthritis has, has gotten worse since I've had that operation. The gap between the, the knee has minimised. So [sighs] when I'm, when I'm walking, I feel, well, I can't actually straighten my leg properly, and when I'm walking, I have to stand in a certain way to try and get my leg to walk. So it, it seems as though it's a quite a complicated issue. They just decided, initially they decided I wasn't old enough to do an operation. TKRB05 (Also coded in Wellbeing and Social engagement- adapting to activities)  During those last few years, the pain in my knee got worse. It was my left knee. Erm, I'm quite swollen, erm, and then further down the line, maybe two years later, er, my right knee started troubling me. Erm, so I-I just assumed it was in that knee as well. I've since been, erm, told by Professor Turner that it may just be that I've, uh, put too much weight on that leg, and I may not need an op on that knee.TKRB07 | Physiotherapy input was not consistent and sessions were disjointed. Participant felt that the exercises were too simple and were not advanced to suit abilities/ previous experience. As such, has done own research into exercise from online resources but unsure if appropriate. Overall, participant felt that physiotherapy was a waste of time.  Physiotherapy was delivered over the phone and as such, it was difficult for the participant to engage. Moreover, they were not offered any additional care after this episode.  Participant had not been referred to physiotherapy despite a desire to engage and despite having been offered a total knee replacement  Physiotherapy exercises viewed/framed as a way to improve post operative outcomes rather than reduce the need for an operation. Exercises experienced as being painful but will help strengthen the muscles and help with recovery as the operation is difficult.  Participant had a number of episodes of physiotherapy input. Viewed physiotherapy as a way to manage the pain and had experienced benefit from previous research studies. However, noted that exercise can be very painful at times. Did experience benefit if they pushed through the pain and this helped with knee flexibility.  Participant had multiple sessions of physiotherapy and was provided resistance exercises. Did not find the exericses helpful or personalised and did not reflect his knowledge of exercise. Did not make a difference to their condition.  Participant experienced lots of input and completed a variety of exercises. Felt that they were helpful, specifically hydrotherapy but that as the knee advanced, they became less effective.  Attended a 6 week exercise group and appreciated that the aim was to help. However, the participant did not enjoy them and felt that they were hurting rather than helping  A total knee replacement was deemed to be the only viable option for the participant following a second opinion with a surgeon  The GP confirmed to the participant that the diagnosis of osteoarthritis warranted a knee replacement  GP/surgeon confirmed that the knee/s needed replacing due to reductions in mobility.  Surgeon recommended a replacement knee and participant was placed on a knee replacement waiting list  GP/surgeon recommended surgery and that there were no other alternatives  GP/surgeon recommended surgery and that there were no other alternatives  Communication from surgeon framed in biomedical language. Understanding of osteoarthritis related to this and although recommending the operation, participant informed not yet old enough.  Surgeon reassured participant that more recent right knee was due to altered biomechanics and may not need an operation. | **Opportunity**  **Opportunity**  **Opportunity**  **Opportunity**  **Opportunity**  **Opportunity**  **Opportunity**  **Opportunity**  **Opportunity**  **Capability**  **Opportunity**  **Capability**  **Opportunity**  **Capability**  **Opportunity**  **Capability**  **Opportunity**  **Capability**  **Opportunity**  **Capability**  **Opportunity**  **Opportunity** |
| --- | --- | --- | --- | --- |
| **Waiting times** | **Theme 2:Theme 2: Interactions with Healthcare Professionals: Focus on a Surgical Solution**  **The experience of waiting for surgery** | Erm, well I was offered, erm, well, I'm saying offered - I was told that it could probably be flushed out but, er, when I saw the, erm, the surgeon a couple of weeks ago, he said it's not an option with mine because it's, it's arthritic bone and it's bone on bone, you know, so it wouldn't do any good. He said all, all that would do is disturb all the debris that's behind my knee and cause more problems. TKRB03 (Also coded in altered health beliefs- bone on bone.  and then it was a friend who remarked, especially during winter when I'm in leggings. Oh, you've gone a bit bowlegged. So I asked both these, er, consultants, both Professor Turner and Mr Riley, and they said it was cartilage wear. Erm, so, er, I had-, I hadn't imagined it. So I - as I say - I am aware I need to like shift my balance a bit, erm, and be conscious, erm, of that. TKRB07  The surgeon looked at the x-rays and said, 'There's nothing I can do apart from replace it.' So...  I was a bit shocked myself because I thought they were just going to, you know - there was a bit of floating bit and then they do this, and he just went, 'There's nothing I can do with that apart from replace it.' TKRB08  Had some more X-rays, which will have been probably 18 months ago now, and, um, said he would put me on the list for knee surgery for a partial replacement, but, um, it could end up as a full replacement, um, you know, once - and that was, that was how it was left, really. Um, so I'm just waiting since then, and I've had no therapy, I've had no treatment. TKRB06 (Also coded to waiting times)  I, I am on a waiting list, but that is a private waiting list. Originally, when all this started, I had a-, an appointment in the January, after the, er, x-ray, and I was, I took a private consultation to, sort of, thinking it would push me forward faster. Um, but then he found out that I had had steroid injections, and said he couldn't do anything for six months anyway. Er, but I'm still on the private waiting list, but, er, at 15 grand, and I've been waiting well over a year now, I said to, they should put me back on the NHS, and when I went in August, the gentleman I saw that day said he couldn't put me on the list, because he couldn't promise to do the operation within the 18 weeks that is the criteria now for knee surgery, because I had anaemia. I was anaemic. Now the anaemi-, the anaemia is sorted out. Um, I have asked to be re-referred, but that was a fortnight ago and I still have no, no date yet to go and see him. TKRB02  Had some more X-rays, which will have been probably 18 months ago now, and, um, said he would put me on the list for knee surgery for a partial replacement, but, um, it could end up as a full replacement, um, you know, once - and that was, that was how it was left, really. Um, so I'm just waiting since then, and I've had no therapy, I've had no treatment. TKRB06 (Also coded to communication with GP/Surgeon)  I was speaking to the person on appointments - they say it's all recorded and stuff like that - and I was really, really annoyed and they said you've only been on here since July last year. So I was thinking, I get - how can I say? - ratty sometimes, and I really spoke out of turn, and I said, 'So are you telling me you don't know when I'm going to get my next appointment, and I'm suffering with all this pain and everything?' I said, 'I might as well do myself in, then, hadn't I? Save you a job of doing it later on?' Which is a stupid thing to say. It was actually a spur-of-the-moment thing, so I was that - they can't give you a definite answer. You're also in this pain, and then you're thinking, they're not even helping at all. They couldn't give you, say, 'Oh, it might be a couple of months,' or, 'It might...' You know, it's something to think about, or something to look forward to. TKR417 (Also coded in wellbeing and social engagement- wellbeing)  and then COVID happened and, erm, I was, erm, hoping to be on a waiting list. Er, and then, as I say, COVID happened, and I couldn't have, er, seen my GP, etcetera, etcetera, or move it forward. During those last few years, the pain in my knee got worse. It was my left knee TKRB07  So it's its just something very difficult, and at times……it just, it gets you down. It does get you down, waiting for, it be fixed, yeah… Oh, it is incredibly frustrating. And, and there are times when it, it does get to you and you feel [sighs] I wouldn’t say to-, depression, but you feel down. You feel down and then you have a better day. TKRB05 (Also coded in Healthcare interactions- waiting lists) | Interaction with the surgeon created negative expectations and influenced perception of condition/ what would help manage pain.  Biomechanical changes framed as a physiological change which participant has little ability to effect. Medical confirmation validated the worry and led to increased body awareness.  Sudden, definitive medical judgment disrupted the expectation of a simpler fix. Reinforces a sense of irreversible damage, which can amplify distress and symptom perception.  Advised to placed on the waiting list with an uncertain treatment plan from the surgeon.  The participant experienced significant delays and poor coordination between private and NHS care. Medical criteria like anaemia affected surgical eligibility, leaving them stuck between systems with no clear pathway forward.  The participant experienced significant delays and uncertain communication with no further input  The participant expressed frustration regarding a lack of clarity whilst waiting for surgery. This resulted in an emotional response and a sense of disappointment due to ongoing pain.  Unable to access care and therefore could not move forward with surgery. Over this time, knee pain worsened  Experienced frustration and low mood whilst waiting for surgery | **Opportunity**  **Capability**  **Opportunity**  **Opportunity**  **Opportunity**  **Opportunity**  **Opportunity**  **Opportunity**  **Motivation**  **Opportunity**  **Opportunity** |

| **Theme 2: Healthcare interactions**  Participants described varied and often contrasting experiences with healthcare professionals involved in the management of knee osteoarthritis. Interactions with surgeons and GPs were frequently framed around the inevitability of surgery, with many participants reporting that total knee replacement (TKR) was presented as the only viable option. This definitive framing often followed imaging or clinical assessments and contributed to a sense of irreversible damage. The language used by clinicians—such as confirmation of “bone-on-bone” contact or reduced joint space—reinforced beliefs about degeneration and validated concerns about worsening symptoms.  The medical framing of osteoarthritis as a biomedical issue led participants to feel that they had limited ability to influence their condition through non-surgical means. This perception was amplified by sudden and definitive judgments from clinicians, which disrupted expectations of simpler fixes and increased emotional distress. Some participants reported that these interactions heightened body awareness and symptom perception, making them more attuned to pain and functional limitations.  Experiences with physiotherapy were mixed. While physiotherapy was generally viewed as acceptable, it was often framed as a short-term intervention to prepare for surgery rather than a tool for long-term management or prevention. This framing limited opportunities for behaviour change and reduced the perceived value of physiotherapy as a standalone treatment.  Several participants described physiotherapy input as inconsistent and disjointed, with exercises that were either too basic or too challenging. A lack of personalisation was a common concern, with some feeling that the exercises did not reflect their prior experience or knowledge of physical activity. In some cases, participants were discharged prematurely due to difficulty with exercises or lack of perceived progress, leaving them unsupported. One participant was only offered telephone calls.  Pain during physiotherapy was a recurring theme. While some participants acknowledged that pushing through pain could lead to improved flexibility, others found it discouraging and a barrier to engagement. A few participants reported benefit from specific modalities such as hydrotherapy, though these were seen as less effective as the condition progressed. In response to dissatisfaction with physiotherapy, some participants turned to online resources to guide their exercise routines. However, this self-directed approach was accompanied by uncertainty about the appropriateness and safety of the exercises.  The participants encountered prolonged delays and poor coordination between NHS and private healthcare services. Medical conditions such as anaemia impacted their surgical eligibility, leaving them without a clear or supported pathway forward. During this time, they experienced emotional distress, frustration, and a decline in mood due to ongoing pain and lack of communication. The inability to access timely care led to worsening knee pain and a sense of disappointment.  Overall, interactions with surgeons, GPs, and physiotherapists shaped participants’ beliefs about their condition and influenced their treatment expectations. The dominant narrative of surgery as inevitable often overshadowed the potential of conservative management, leaving participants feeling passive in their care and uncertain about alternative options. Waiting for surgery results in worsened knee pain and disappointment with support.. | | | | |
| --- | --- | --- | --- | --- |
|  | | | | |
| **Theme 3-** **The Emotional Burden of Living with KOA** | | | | |
| **Codes**  **Adapting to the condition**  **Wellbeing**  **Sleep** | SUB-THEME 1- Valued Social Engagement Amid Physical Constraints  Participants attempts to remain active and socially engaged whilst managing their condition and pain experience  Theme 3: The Emotional Burden of Living with KOA  Participants experience of pain, loss of independence, social engagement. and sense of self. Related to mood, quality of life and outlook for the future. Experienced whilst navigating the health system and looking for a solution to their pain.  SUB-THEME 2- Pain-Related Sleep Disruption and Its Ripple Effects  Participants experience of the effect of their condition on their sleep and sleep quality, including night related pain | I think in relation to seeing a lot of other people hob- hobbling around, erm, I don't think mine's that bad really. You know, when you see people having difficulty walking and walking with, er, mobility aids, etc.  , I can probably walk about, erm, probably about 300 or 400 yards and then we have to stop, erm, and then I start again sort of. And then, but I, I do, do, erm, I play bowls five times a week. Er, but then on the morning afterwards, on the side, on the inside of my leg, er, above, above the knee on the inside, it's awful there, er, it aches terribly and I do get cramp there. But as I say, I do play five times a week, but that's sort of a general walking exercise really, because you walk, you, you send the bowl and then you, you can wait about half - another minute, erm, before, before you have to send another bowl, so you do get rest in between. It's not a continual three quarters of an hour movement. TKRB01  And since then, which will be about, oh, eight years ago, nine years ago, it's gradually got worse to the point where, you know, I'm really struggling. Not so much just sitting around but if, if I'm doing any walking, especially slow walking around the shops or just wondering about, then that tends to make it worse, as does just standing...So, you know, I try to, to sit. TKR04 (Also coded in – Coping strategies- avoidance)  And they thought it was a, a meniscus tear, and they did operate on that initially, but said that it was too, um, it was frayed. They couldn't put it back together and there was a ramp lesion that they didn't see the point of fixing. So. But in this process, they did notice that there was osteoarthritis. So, so the osteoarthritis has, has gotten worse since I've had that operation. The gap between the, the knee has minimised. So [sighs] when I'm, when I'm walking, I feel, well, I can't actually straighten my leg properly, and when I'm walking, I have to stand in a certain way to try and get my leg to walk. So it, it seems as though it's a quite a complicated issue. They just decided, initially they decided I wasn't old enough to do an operation. TKRB05 (Also coded in Healthcare Interactions- communication with GP/ Surgeon)  Initially - I do yeah. In-, initially I was, I was coming down the stairs on my backside. You know, and then going up. Luckily there are two, there are railings on both sides, because I rent, rent the house, the stairs, stairs are very steep. But I was, I'm able to pull myself up using my arms as well, you know, so I'm not putting too much pressure, but coming down, um, was on my backside. And then I, then I learned to crab walk, and, and now I'm practising trying to walk straight, you know, with my feet straight as opposed to sideways TKRB05  Erm, so, er, my world closed down rather quite a lot. Me and my partner are - were big walkers, always have been. Erm, so from doing ten miles on a walk throughout a day, you know, with breaks, erm, that got worse and worse to where I'm, I was down to two miles during all this period. TKRB07  Erm, I've al-, I'm, I'm still active, and I've always been active. Er, what the, the knee has done, erm, it's obviously curtailed to a large extent walking. Erm, I do a lot of outdoor activities and, and of course, it's, it's impinged on that. Erm, the pain in the last couple of years has been really, really, er, demoralising. Erm, and obviously there are certain things that I can no longer do, or I can - I try to, to, to live as normal a life as possible, so I know it's sore when I do certain things, but certain things have to be done, so I just do them anyway….Erm, for example, I can't, I can't go fishing on rivers anymore because I can't walk the distances that you need to walk to get there, and I can't stand all day as I used to do when I was fishing and, and that is a real pain, that, because I, I used to love fishing the rivers. Er, everything has to be done sat down now. TKRB10 (Also coded in wellbeing and social engagement- wellbeing)  Mm. I mean, we go on holiday to warmer climates because that does help a little bit. But I end up meself and me partner - like, he loves walking. We used to both love walking. So he'll go off walking and that's absolutely fine but I can't do that. I have to wait till he comes back. Yes. It is. And going up and downstairs. I can't do that. It takes me ages, ages to go upstairs and, and to come down. You can't, you can't go shopping 'cause you can't walk that far but if you carry your bags…you can't carry your bags and your walking stick and keep your balance…I mean, I've got four children and I've got six grandchildren.  And they do run around and they get you whatever you want. But that's - like you say, it stops you moving, which is not a good thing…One of them plays football and if I go to play football - watch him play football, I'll end up in the car halfway through 'cause I can't stand up. TKRB10 (Also coded in coping strategies- avoidance)  Well, I can just tell you it's affected my lifestyle a lot. It's closed my, erm… As a final summary from me… It's closed my world down. Very much so. Erm, I still drive, but I couldn't drive a great big distance, and maybe, er, it's time to think about having a-, an automatic car. It, it hurts you. But I had an automatic car decades ago and didn't like it, er, but I'll have to get over that. Hmm. My partner said that. He's got an automatic. Erm, and he said, 'Maybe it's time you… - See how you go after your op.' 'But maybe it's time, er, to not have to use gears so much now. Erm, but it has closed my world down. Erm, but I… Not… My friends are walkers, so I struggle to keep up with. I don't go in big groups. I don't do that, but I go with different friends…Just the two of us. I, I struggle to do that, erm, what I have to do is do nothing really much at home before I go for the walk because walking about doing jobs that… I have to pay for pace meself. So I won't stand and iron or hoover and clean before I go for a walk because then I can't do the walk. And that walk might just be, I don't know, a couple of miles with a sit-down. TKRB07  And then it's difficult to, to turn over in bed, I have to put my leg out straight and then roll over on it that way as opposed to trying to bend it and move over, so it's difficult to turn over. I've got used to putting my legs straight and turn over that way. TKRB01  Erm, and I perhaps sometimes I have to stop. I can maybe walk with the naproxen a couple of miles, but I have to keep having a sit-down TKRB07 (Also coded in coping strategies- pharmacology)  Er, and I, I jokingly said to the doctor that I saw, the first doctor, er, first consultant that, 'I'm gonna end up in a nursing home at this rate, because it's taking so long.' And he just said not to be so silly. Er, but it's be-, it's been a year, and I'm still messing about, and I said the only other alternative is to throw myself down stairs. TKRB02  And I said, and I just said to him, 'Well, I'm sorry, but it's taken my whole quality of life. I can't do anything.' I'm a keen outdoor person. I, I like gardening and everything, so it's stopped me doing any of that. TKRB03  I was speaking to the person on appointments - they say it's all recorded and stuff like that - and I was really, really annoyed and they said you've only been on here since July last year. So I was thinking, I get - how can I say? - ratty sometimes, and I really spoke out of turn, and I said, 'So are you telling me you don't know when I'm going to get my next appointment, and I'm suffering with all this pain and everything?' I said, 'I might as well do myself in, then, hadn't I? Save you a job of doing it later on?' Which is a stupid thing to say. It was actually a spur-of-the-moment thing, so I was that - they can't give you a definite answer. You're also in this pain, and then you're thinking, they're not even helping at all. They couldn't give you, say, 'Oh, it might be a couple of months,' or, 'It might...' You know, it's something to think about, or something to look forward to. TKR417 (Also coded in healthcare interactions- waiting lists)  I've just finish going to the gym, I've been going to the gym for a number of years and it's mainly and re-, and more recently, it's mainly been sort of upper body exercises rather than leg, and ev-, everything that puts a strain on it, but er, I d-, I don't really go anymore because it was, was getting really painful, and again, there may be some point in the future where you know, I can feel comfortable about going back again, because I do miss it. TKRB04 (Also coded in – Coping strategies- avoidance)  And in, in the time that I've had this, I have seen quite a decline in my ability to walk. Previous to the injury, I was going to the gym, I was going to yoga. I was riding my bike, I was going on walks. I. I was very fit and able. And then I got the injury and it just, it, it just impacted my life in such a way that I would never have imagined. I wasn't able to do things, getting up the stairs, getting down the stairs, just walking. Just walking was, was difficult. And it just progressed to the, to the degree that I'm using a, a crutch to help me. TKRB05 (Also coded in – Coping strategies- avoidance)  Erm, I've al-, I'm, I'm still active, and I've always been active. Er, what the, the knee has done, erm, it's obviously curtailed to a large extent walking. Erm, I do a lot of outdoor activities and, and of course, it's, it's impinged on that. Erm, the pain in the last couple of years has been really, really, er, demoralising. Erm, and obviously there are certain things that I can no longer do, or I can - I try to, to, to live as normal a life as possible, so I know it's sore when I do certain things, but certain things have to be done, so I just do them anyway….Erm, for example, I can't, I can't go fishing on rivers anymore because I can't walk the distances that you need to walk to get there, and I can't stand all day as I used to do when I was fishing and, and that is a real pain, that, because I, I used to love fishing the rivers. Er, everything has to be done sat down now. TKRB10 (Also coded in Wellbeing and social engagement- Adapting to the condition)  So it's its just something very difficult, and at times……it just, it gets you down. It does get you down, waiting for, it be fixed, yeah… Oh, it is incredibly frustrating. And, and there are times when it, it does get to you and you feel [sighs] I wouldn’t say to-, depression, but you feel down. You feel down and then you have a better day. TKRB05 (Also coded in Healthcare interactions- waiting lists)  Oh, thank you. I think my mental health an' all, er, just to put that into the, er, bag of goodies. It's affected my mental health. I, I, I, I don't know if it was COVID as well that started it. You know, er, the lockdowns, etc. It's affected everybody, but, er my knee… Now I'm out and about, er, when all that finished and I was vaccinated right through it, er, I still find my mental health-… I've changed.It's affected my personality  Erm, the constant pain. It makes me very crabby at the end of the day, erm…Erm, and not very participant and tolerant. I really have to think about, er, you know, having been a teacher and always having patience and tolerance. And, er, you know, prepared to listen. Erm, er, I, I do find it's affected me as a person. TKRB07  Definitely, definitely is. It has a massive impact, really, and it has a massive impact on your mental health. It - I mean, I'm only 65, 64. So really, I shouldn't be like this. I should be…Another 20 years, 15 years, I should be like this. And it's awful. TKRB12  There's some, there's some worries related to the operation. There's some worries related to the operation, yeah. Low at times, whilst waiting. But as I said, I’m wanting it now. I want to get on. TKR416  but certainly no night pain, no, no I don't, and I've got, I've got, if I, if I, if I do I then take a couple of paracetamol at night and that... that eases it. TKRB04 (Also coded in Coping strategies- pharmacology)  So it, it like pulses and, and I've had to take stronger co-codamol at night, but I'm not a person that wants to take tablets…So, you know, I'm, I'm very… Health conscious. No, what I, what I do is if I feel in the morning, before I'm taking the dog out, if I feel I'm in pain I will take two of the 8mgs, um, and then I will try the rest of the day to go without it. And then when I sleep, I'll take two 15 mgs. But I do find in the morning, when I've ta-, taken those, that my head is like cotton wool. TKRB05 (Also coded in Coping strategies- pharmacology)  Um, not very often. I use one of these - you know the, the little v-pillow that you put between your knees?  I find that that has been a really good release, because, um, it keeps me hips in line as well, and I sleep on my - I do tend to sleep on my left-hand side most of the time. Obviously, with this back trauma, it's been a little bit different lately. But I have found that is, is very good, because it did used to wake me up. The pain, you know, when I put one knee over the other. And sometimes, if I turn - you know, we all turn a strange way sometimes, and it's like, whoa! You know? That hurts. TKRB06  It, it seems to be more, er, night, nighttime when I, when I'm - when you think that you'd be resting and everything would be okay, the, the pain that then comes back during the night, and it, it - that can have an effect because then you've got sleep deprivation because it, it is so painful, and you can't get comfortable and that sort of thing, you know. Er, and I think it's that that then sometimes can accumulate and it's more like through the lack of sleep and this, that and the other, that it's got more effects on your health, I think, than the actual pain side of it.. if you have a couple of bad nights and you're not getting any sleep, then that, that does - obviously, as we all know, sleep deprivation doesn't, doesn't help at all…I think if they can find ways to, to, you know, reduce the discomfort, I think someone's, you know, everyday life will improve massively because they'll sleep better, they'll feel better within themselves and stuff like that, you know.TKRB08  I'm up at night. I get up at night now, so I don't really sleep through the night 'cause if you turn over fully, you crack your knee. So then you're up. TKRB12  I mean I just feel low at times. I think my sleep is the worst part. If I turn over. It wakes me. TKR513 | Relating own condition to others and although has adapted, is not as bad. Recognising the importance of staying active and finding activities that are manageable and incorporate rest breaks. This is in the context of ongoing pain.  Continues to attempt to remain active but requires more rest periods to manage the condition.  Participant is attempting to stay active but has had to adapt the biomechanics of walking to achieve this. Framing this change alongside the complexity of the condition.  Participant adapting to condition by using different methods to ascend/descend stairs. Sense of independent trial and error as they navigate the conditions impact.  Participant describes the desire to keep walking but has to reduce the distance significantly due to condition  Participant describing a desire to remain active but recognising that in order to this has had to adapt expectations and manage pain through sitting, rather than standing.  Attempting to engage in valued activities such as going on holiday or watching grandchildren play sport. However, has had to adapt these, reducing activity levels and noticing the associated challenges with this.  Participant reflects on how OA has limited ability to socialise, and interact with others. Has had to adapt walking and is considering adapting her car. Recognising the need to pace activities to achieve social engagement  Participant changed the way they sleep in order to reduce the risk of pain at night.  Participant now has to stop when walking and rest and/or reduce their mileage  Participant using humour to expressing distress in relation to long wait and sense of not being taken seriously.    Participant experiencing and expressing distress in relation to change in quality of life and hobbies.  Participant experiencing and expressing distress in relation to long wait and sense of not being taken seriously.  Participant unable to continue with attending the gym which they enjoy. Is hopeful for the future.  Participant experienced a decline in engagement in activities that brought enjoyment and in physical health. Describes how condition has impacted life to a degree that is beyond their comprehension.  Participant expressed being disheartened by pain and subsequent reduction in activities and change in lifestyle  Participant experienced feeling down whilst waiting for surgery. They felt frustrated and low in mood  Participant expressed that their mental health has been affected by their knee pain. They describe how the constant pain has reduced their tolerance and affected their personality/ them as a person.  Participant expressed the significant negative impact that pain has had on their mental health  Participant expressed anxiety in the context of the operation and reports feeling low whilst waiting for surgery as they want to move on.  Participant denies night pain and if they do experience night pain, it is manageable with paracetemol  Although participant is health conscious and reluctant to take medication, finds that in order to sleep well and manage pain, takes co-codamol with morning side effects  Participant finds that overall, sleep is no longer disturbed as they use a pillow. However, has experienced night pain and being woken, especially when turning over.  Participant describes that night related pain is unusual as should be pain free when rested. However, notes that experiencing night pain which effects sleep is common and due to this, every day life becomes challenging.  Participant reports a lack of sleep due to pain.  Participants reports feeling low due to poor sleep related to pain | **Motivation**  **Motivation**  **Behaviour**  **Motivation**  **Opportunity**  **Motivation**  **Behaviour**  **Motivation**  **Motivation**  **Behaviour**  **Motivation**  **Behaviour**  **Motivation**  **Behaviour**  **Motivation**  **Behaviour**  **Motivation**  **Behaviour**  **Motivation**  **Motivation**  **Motivation**  **Opportunity**  **Motivation**  **Behaviour**  **Motivation**  **Behaviour**  **Motivation**  **Motivation**  **Motivation**  **Motivation**  **Motivation**  **Motivation**  **Behaviour**  **Motivation**  **Motivation**  **Motivation**  **Motivation**  **Motivation** |

| **Theme 3 summary:**  Participants described a complex interplay between pain, activity adaptation, and emotional wellbeing. Despite ongoing pain, many expressed a strong desire to remain active and socially engaged, though this often required significant adjustments.  Participants consistently demonstrated efforts to stay active, often modifying how they walk, ascend/descend stairs, or engage in daily routines. These adaptations were framed as necessary responses to the complexity of their condition. Activities such as going on holiday or watching grandchildren play sport were still pursued, albeit with reduced intensity and increased planning around rest breaks.  The condition was reported to limit social interaction and independence. Some participants reflected on the need to pace activities to maintain social engagement, including considering adaptations to their vehicles. There was a sense of trial and error in navigating these limitations, with participants striving to preserve valued aspects of life despite physical constraints.  The emotional toll of the condition was evident. Participants expressed feelings of frustration, sadness, and anxiety—particularly in relation to long waiting times for surgery and the perceived lack of seriousness with which their concerns were treated. Several described a decline in mood and mental health, noting changes in personality and emotional resilience due to persistent pain.  Sleep disturbances were commonly reported, often linked to night pain. While some participants found relief through medication or positional adjustments (e.g., using a pillow), others described poor sleep as a significant contributor to emotional distress and reduced quality of life. The impact of disrupted sleep extended into daily functioning and mood.  Despite challenges, some participants maintained a comparative perspective, noting that their condition was not as severe as others. This framing often coexisted with a health-conscious mindset and a reluctance to rely on medication, though many acknowledged its role in maintaining function and sleep quality. | | | | |
| --- | --- | --- | --- | --- |
|  | | | | |
| **Theme 4:** **Coping Strategies for Managing KOA Pain** | | | | |
| **Codes**  **Orthotics**  **Avoidance**  **Pharmacology**  **Corticosteroid Injection** | SUB-THEME 2- Pain Relief Strategies  The use of braces to improve pain and support the knee  SUB-THEME 1- Adapt or Avoid  The strategy of avoiding activities or reducing activities in order to manage pain.  SUB-THEME 2- Pain Relief Strategies  The use of medication such as paracetamol, ibuprofen or opiates to reduce pain and manage the condition  SUB-THEME 2- Pain Relief Strategies  The use of steroid injections to manage pain | I've tried wearing, er, support braces and, erm, the elasticated version of support braces and also the ones that have got support down the side of them. Er, but it doesn't, it doesn't make any difference really and the elasticated ones, er, tend to make my legs swell up anyway, you know, 'cause, 'cause the one I've, I've got is elasticated and then there's also a strap on it which you wrap around about three times, er, below and above the knee so that, when I was wearing it, it was making my leg swell up somewhat.TKRB01  Yeah, I've, I've, yeah, I wear a knee brace. Again, if I know that I'm going to be going out walking around for any length... I don't wear a knee brace when I'm in the house, but if I'm going out, I put a sleeve on, on the knee. Yes, er, yes, yeah it helps the pain, and, and and it's also, it warms the knee as well. TKRB04  Oh, no. No, um, little or nothing. I, I couldn't walk to the end of the street and back. I have to go in the car, and, because of the knee and the shoulder, that's getting more difficult. TKRB02  And since then, which will be about, oh, eight years ago, nine years ago, it's gradually got worse to the point where, you know, I'm really struggling. Not so much just sitting around but if, if I'm doing any walking, especially slow walking around the shops or just wondering about, then that tends to make it worse, as does just standing...So, you know, I try to, to sit. TKRB04 (Also coded in wellbeing and social engagement- wellbeing)  I've just finish going to the gym, I've been going to the gym for a number of years and it's mainly and re-, and more recently, it's mainly been sort of upper body exercises rather than leg, and ev-, everything that puts a strain on it, but er, I d-, I don't really go anymore because it was, was getting really painful, and again, there may be some point in the future where you know, I can feel comfortable about going back again, because I do miss it. TKRB04 (Also coded in wellbeing and social engagement- wellbeing)  And in, in the time that I've had this, I have seen quite a decline in my ability to walk. Previous to the injury, I was going to the gym, I was going to yoga. I was riding my bike, I was going on walks. I. I was very fit and able. And then I got the injury and it just, it, it just impacted my life in such a way that I would never have imagined. I wasn't able to do things, getting up the stairs, getting down the stairs, just walking. Just walking was, was difficult. And it just progressed to the, to the degree that I'm using a, a crutch to help me. TKRB05 (Also coded in – wellbeing and social engagement- wellbeing)  You know, I can't walk around the Trafford Centre, because that would be too much for me. You know, it would start like, you know, my steel drum band would start again, um, so I don't do that. So I don't drive far, um, so I don't have the experience of if I did. I'll tell you what, I got stuck in some traffic for 45 minutes, and I, as, as I got out of the car, I had to stand there for about three minutes, in order for my leg to straighten out. TKRB05 (Also coded in wellbeing and social engagement- adapting to condition)  Mm. I mean, we go on holiday to warmer climates because that does help a little bit. But I end up meself and me partner - like, he loves walking. We used to both love walking. So he'll go off walking and that's absolutely fine but I can't do that. I have to wait till he comes back. Yes. It is. And going up and downstairs. I can't do that. It takes me ages, ages to go upstairs and, and to come down. You can't, you can't go shopping 'cause you can't walk that far but if you carry your bags…you can't carry your bags and your walking stick and keep your balance…I mean, I've got four children and I've got six grandchildren.  And they do run around and they get you whatever you want. But that's - like you say, it stops you moving, which is not a good thing…One of them plays football and if I go to play football - watch him play football, I'll end up in the car halfway through 'cause I can't stand up. TKRB10 (Also coded in wellbeing and social engagement- adapting to condition)  I can't do an evening walk anymore like we'd go out and walk for maybe 20 minutes in an evening, three times a week. Erm, you know, even when it's winter, and it's d-, dark. I can't do that because come two o'clock in the afternoon, it hurts too much, you know. And then in the evening. I've no chance. I just need to sit down, to be honest TKRB07  Erm, then I have, then I have a painkiller (Codydramol) first thing in the morning and then, erm, by the time I've moved around a bit, the, the pain's easier. So whether or not that's the pill or just the physical, er, activity of moving, you know, is helping. TKRB01  Er, my doctor put me on co-dydramol, er, and I was in so much discomfort that I started taking ibuprofen, as well, but my stomach's not in good condition, and, er, I really shouldn't have been taking them, but I'm managing, at the moment. I'm taking two co-dydramol in the morning, and two, um, ibuprofen, and at, sort of, four/five o'clock, round the early teatime. TKRB02  it doesn't matter what, erm, you know, er, painkillers I have, it doesn't relieve it at all TKRB03  no, no I don't, and I've got, I've got, if I, if I, if I do I then take a couple of paracetamol at night and that that eases it TKRB04 (Also coded in Wellbeing and social engagement- wellbeing)  I'm just taking co-codamol at the moment, for it. TKRB05  Oh, it (paracetamol) doesn't work No, no. Neither does ibuprofen, you know…Over-the-counter. Erm…  Naproxen. TKRB07  Generally co-dydramol. If, if that's what they are, yeah. I have taken, I've taken them (Paracetemol), erm, basically - basically, er, on a basis of as and when they're needed. They're, they're prescribed for me to be taken four times a day - two tablets four times a day. I think they're 500 milligram. TKRB09  Voltarol, etc., etc. And, and painkillers. And, and quite honestly, the o-, the only thing that I've found - I was never one for taking tablets and, erm, I, I would never take painkillers as a preventative medicine. Er, I always waited until it was quite unbearable, then perhaps I would take one and, of course, it, it, it didn't work as good as it was meant to work. Erm, so now, in the past, I would say, five, six weeks, er, when I've got up in the morning, I've taken, er, a tramadol painkiller. Erm, and I, I, I do, I do believe it's helped. TKR010  So I'm on massive amounts of painkillers all the time. Erm…I, I have, er, Zapain and I've got the Oramorph. So I take them. I start…You get constipation…which is not very good. Yes, you do. I have to take this, er - or the laxative TKRB12  I first got the pain, and then since then, I've been able to cope with the pain with different creams on the knee, and paracetamol, but of late it seems to have really gone worse. TKR417  So it, it like pulses and, and I've had to take stronger co-codamol at night, but I'm not a person that wants to take tablets…So, you know, I'm, I'm very… Health conscious. No, what I, what I do is if I feel in the morning, before I'm taking the dog out, if I feel I'm in pain I will take two of the 8mgs, um, and then I will try the rest of the day to go without it. And then when I sleep, I'll take two 15 mgs. But I do find in the morning, when I've ta-, taken those, that my head is like cotton wool. TKRB05 (Also coded in wellbeing and social engagement- sleep)  Erm, and I perhaps sometimes I have to stop. I can maybe walk with the naproxen a couple of miles, but I have to keep having a sit-down TKRB07 (Also coded in Wellbeing and social engagement- adapting to the condition)  Yeah, steroid, er, cortisone. Yeah, yeah. I've had, er, two injections in my knee, which, erm, haven't, basically haven't made any difference. Although I've had an injection in my thumb and, er, that's helped. But it, it hasn't made any difference to the knee. TKRB01  Um, but I, then again, I, I was told that steroids would be like a miracle to me, and they did absolutely nothing. They just made it…the pain worse. TKRB02  and the, the steroid injection last June which really didn't make a massive difference. TKRB04  Erm, no, no, I've, I've also had a number of, erm, is it the steroid injections, into the knee. Yeah. I've had quite a few of them, I've had about six of them. Erm, no, it's about every, every 12 months.  It did at first, to, to be honest with you, the first ones, erm, gave an awful lot of relief, but then gradually over time it, it's waned and it - the last one didn't really, it didn't even last a week, to be honest with you, the last one. TKRB08  prior to COVID for some years I was having cortisone injections…In my left knee. It was my GP who said around the January time, just before we had lockdowns, you really need to get on a waiting list now because the gap between injections with pain relief was getting less and less. So instead of, like, it lasting me four months and a bit longer in some cases between, erm, cortisone, it was only lasting the six to eight weeks. Er, I did know that my GP would say this to me because some years ago I had bad arthritis in my foot, and I had an op at Stepping Hill for that, er, because the injections were getting less and less, er, successful. TKRB07 | Participant has tried elasticated and rigid braces but didn’t experience any benefit. Also reported adverse effects such as swelling.  Participant used a brace when walking further and found this of benefit.  Participant has stopped walking due to their pain  Participant sits more to reduce the impact of their pain  Participant adapted the gym, avoiding leg exercises. Now has stopped going to the gym due to pain. However, is hopeful of returning to the gym in the future.  Participant now uses a crutch to reduce the impact of walking and reports struggling to do daily activities.  Participant has stopped walking around the shops and drives less in order to avoid pain or stiffness.  Participant has stopped engaging in walking, stairs, carrying due to the time and effort related to the activity.  Due to the pain, participant has stopped walking for pleasure and is now sitting more  Participant’s pain is improved with codydramol taken in the morning. Also recognizes the benefit of movement/ exercise  Participant takes codydramol and ibuprofen to manage pain. Recognises the side effects of pain relief but able to manage.  Participant has tried pain relief but is unable to manage pain  Generally manages ok without pain relief. If does have pain, it will be at night and will then take paracetemol  Participant take co-codamol for their pain  Participant reflects that paracetamol and ibuprofen don’t help with pain.Has also tried naproxen.  Participant takes co-dydramol and paracetamol to manage the pain.  Participant is releuctant to take medication but had tried voltarol, paracetamol without much success. Notes that tramadol is the most effective.  Participant takes a variety of painkillers including strong opiates. Recognises the side effects of constipation related to pain relief.  Participant has coped with paracetamol and topical creams but is finding they are becoming less effective.  Participant is reluctant to take medication however finds that co-codamol is helpful. Recognises side effects, especially morning grogginess.  Participant uses naproxen to help with exercise  Although participant had previously benefitted from a steroid injection. Does not feel they help knee pain  Participant had high expectations of steroids but there was no benefit, in fact, they made the pain worse.  Participant did not benefit from a steroid injection  Participant has had multiple injections andalthough previously benefitted, recently they have become ineffective.  Participant has had multiple injections and although previously benefitted, recently they have become ineffective. | **Behaviour**  **Behaviour**  **Behaviour**  **Motivation**  **Behaviour**  **Motivation**  **Behaviour**  **Motivation**  **Behaviour**  **Motivation**  **Behaviour**  **Motivation**  **Behaviour**  **Motivation**  **Behaviour**  **Behaviour**  **Behaviour**  **Behaviour**  **Behaviour**  **Behaviour**  **Behaviour**  **Behaviour**  **Behaviour**  **Behaviour**  **Behaviour**  **Behaviour**  **Behaviour**  **Behaviour**  **Behaviour**  **Motivation**  **Behaviour**  **Behaviour**  **Behaviour** |
| **Theme 4 summary**  Participants reported a range of strategies to manage their pain and maintain daily functioning, with varying degrees of success.  Several participants experimented with elasticated and rigid braces, though most did not experience significant benefit and some reported adverse effects such as swelling. Braces were occasionally used for longer walks, which provided some relief. Over time, many participants reduced or ceased walking altogether due to pain, opting instead to sit more frequently. The use of crutches was adopted by some to mitigate walking-related discomfort, though this was accompanied by increased difficulty in performing daily activities.  Participants commonly adapted or avoided physical activities. Gym routines were modified to exclude leg exercises, and some eventually stopped attending altogether due to pain, though there was expressed hope of returning in the future. Walking for pleasure, navigating stairs, shopping, and driving were all activities that participants reduced or ceased, often citing pain, stiffness, or the effort required.  Pain relief strategies varied widely. Common medications included paracetamol, ibuprofen, co-codamol, co-dydramol, naproxen, and tramadol. While some participants found these helpful, others reported limited effectiveness or adverse side effects such as grogginess and constipation. A few participants expressed reluctance to rely on medication but acknowledged its necessity. Codydramol was noted as particularly effective when taken in the morning, and movement/exercise was also recognized as beneficial by some.  Experiences with steroid injections were mixed. While some participants had previously benefitted, others reported no improvement or worsening of symptoms. Repeated injections were described as increasingly ineffective over time, despite initial positive outcomes. | | | | |
